# An EZH2 blocker sensitizes histone mutated diffuse midline glioma to cholesterol metabolism inhibitors through an off-target effect

**DOI:** 10.1093/noajnl/vdac018

**Published:** 2022-03-01

**Authors:** Farah Rahal, Caroline Capdevielle, Benoit Rousseau, Julien Izotte, Jean-William Dupuy, David Cappellen, Guillaume Chotard, Mélissa Ménard, Justine Charpentier, Vincent Jecko, Charline Caumont, Edouard Gimbert, Christophe F Grosset, Martin Hagedorn

**Affiliations:** 1 Univ Bordeaux, Campus de Carreire/Victoire, Sciences de la santé/Sciences de l’Homme, Bordeaux, Cedex, France; 2 Inserm U1035, University Bordeaux, Bordeaux, Cedex, France; 3 Animalerie A2, University of Bordeaux, Bordeaux, Cedex, France; 4 Univ. Bordeaux, Plateforme Protéome, Bordeaux, Cedex, France; 5 Department of Neurosurgery, Hôpital Pellegrin, Bordeaux University Hospital, place Amélie Raba Léon, Bordeaux, Cedex, France; 6 Department of Pathology, Hôpital Pellegrin, Bordeaux University Hospital, place Amélie Raba Léon, Bordeaux, Cedex, France

**Keywords:** atorvastatin, cholesterol metabolism, DMG, diffuse midline glioma, EZH2, enhancer Of Zeste homolog 2, H3K27M-mutant, GSK126

## Abstract

**Background:**

Diffuse Midline Glioma, H3K27M-mutant (DMG) is a rare, highly aggressive pediatric tumor affecting the brainstem, and is one of the deadliest cancers. Currently available treatment options such as chemotherapy and radiotherapy do only modestly prolong survival. In this pathology, H3K27 mutations deregulate Polycomb Repressive Complex 2 (PRC2), including enzymatic activity of EZH2, which is therefore under investigation as a therapeutic target.

**Methods:**

We used a chemical EZH2 inhibitor, GSK126, small interfering RNAs, and a CRISPR/Cas9 knockout approaches in a series of DMG tumor cell lines to investigate metabolic treatment responses by proteomic analysis. A combination strategy was elaborated and studied in primary and established DMG cells, spheroid 3D cultures, and *in vivo* in a chick chorio-allantoic membrane DMG assay and an orthotopic intracranial DMG mouse model.

**Results:**

GSK126 shows significant (*P* < .05–.001) inhibitory effects in *in vitro* cell proliferation assays and induces apoptosis. Chemical targeting of EZH2 induced expression of proteins implicated in cholesterol metabolism. Low-dose GSK126 treatment together with statins revealed strong growth inhibition in combinatorial treatments, but not in single treatments, both in DMG cells *in vitro*, in DMG spheroid cultures, and in chick and mouse *in vivo* models (*P* < .05). All statistical tests were two-sided.

**Conclusions:**

Our results reveal an unexpected GSK126-inducible sensitivity to cholesterol biosynthesis inhibitors in highly aggressive pediatric glioma that warrants further evaluation as treatment strategy. This combinatorial therapy should have few side effects because of the low doses used to achieve significant anti-tumor activity.

**Key Points**

- Diffuse midline glioma are sensitive to EZH2 inhibitor GSK126.
- Low-dose GSK126 induces sensitivity of tumor cells to statins.
- GSK126 with Atorvastatin reduces tumor cell growth at low micro molar doses.

**Importance of the Study**

The here described experimental therapy for diffuse midline glioma is a novel form of anti-cancer treatment where one drug which targets EZH2 (GSK126) and other not identified targets renders tumor cells dependent of cholesterol biosynthesis at a low micromolar dose. The combination of the EZH2 targeting drug with a statin, in this case Atorvastatin, which is a well characterized drug, have strong anti-cancer effects at low micromolar doses and is therefore an ideal combination for pediatric patients. This combinatory therapy will most probably have very few toxicities compared to standard chemotherapy, it is patented and clinical trials, also in adult common cancer types are planned.

Identifying novel treatment concepts for incurable cancers remains a major challenge in medicine. Cancer-type specific drugs or so called “precision medicine” are very costly and are available only for some adult cancers. Research for therapies tailored for specific pediatric tumors is still rare,^1^ therefore therapy options are often limited to chemotherapy and radiotherapy with severe side effects.

Drugs targeting proteins regulating epigenetic processes (epi-drugs) have gained increasing attention as treatment for hematological and solid malignancies, including DMG.^2–4^

One key element in organization of the genome are histones and several histone mutations have been discovered that deeply influence transcriptional activity.^5^ For example, reduction of histone methylation by Lysine to Methionine mutations at specific histones (K-to-M at H3K36, H3F3A, or HIST1H3B) has profound effects on cell growth through transcriptional modifications, a phenomenon conserved in all living systems, from plants^6^ to eukaryotic cell, including cancer cells.^7^ Histone mutations alter functioning of effector protein complexes such as PRC2, which includes the methyltransferase EZH2.

Abnormal epigenetic regulation in DMG tumors are a novel therapeutic target, with possibility for pharmacologic intervention to normalize gene expression, most likely by lowering oncogenic protein levels or augmenting tumor suppressors. We have shown that the pan-HDAC inhibitor panobinostat profoundly changes protein expression profiles, leading to overexpression of only two proteins, IRSp53 and EBP50. These two proteins seem to be markers of resistance, since they are required for DMG cell growth.^4^

Interfering with histone methylation, acetylation, or activity of BET domain proteins such as BRD4 are therefore promising strategies against DMG. Several of them are actually studied in clinical DMG trials such as panobinostat, vorinostat, or valproate (for review see^8^)

Analyzing reaction to treatment on a cellular level using high throughput approaches such as microarrays and proteomics combined with diverse artificial intelligence techniques has widely broadened our general understanding of drug mechanisms of action^9^ and can give information on novel potential targets of existing drugs.^10^

In this paper, we analyze the role of cholesterol biosynthesis-related proteins which we found significantly upregulated in DMG cells by GSK126 treatment. We, therefore, hypothesized that EZH2 inhibition by GSK126 renders DMG glioma cells dependent on cholesterol biosynthesis, thus giving the possibility to explore statins or other lipid reducing drugs as treatment option in this pathology. Drug repurposing has already provided novel perspectives for various diseases, including cancer.^11,12^

We provide evidence that simultaneous use of low micromolar doses of GSK126 together with statins, especially Atorvastatin, has significant anti-tumor effects in highly malignant glioma *in vitro* and *in vivo*, thereby opening a novel treatment perspective for this disease with dismal prognosis.

## Methods

### Microarray Analysis

The GSE50021 expression profile (35 DMG samples, 10 normal brain samples), was extracted from the Gene Expression Omnibus database. Raw reads were quantile normalized and log2 transformed. Expression values identified by ILMN_1652913 probe for EZH2 were extracted and analyzed using GraphPad Prism.

### DMG Cell Lines and Primary BXdmg1 Cells

DMG cell lines used in this study were NEM157i, NEM157i-VEGF, NEM163i, SU-DIPG-IVi, SU-DIPG-IVi-Luc and freshly isolated cells from a biopsy termed BXdmg1. Origin and lentiviral modification procedures (including immortalization), as well as culture conditions, have been described elsewhere^4^ and in Supplementary Data. BXdmg1 cells are available from the corresponding author depending on reasonable requests.

### Chemical Inhibitors

EZH2 inhibitor GSK126, Atorvastatin, ACSS2 inhibitor, and Terbinafine have been used (Selleckchemicals, Houston, USA).

### Western Blots

Blots were performed using standard techniques. Details are in the Supplementary Methods section.

### Histone Extraction

Histone extraction was performed according to manufacturer’s instructions (Histone Extraction Kit—ab113476, Abcam, Paris, France).

### Proliferation Assays

Cell growth was measured with the In vitro Toxicology Assay kit (Sulforhodamine B, Sigma Aldrich, Saint Quentin Fallavier, France) according to manufacturer’s instructions. Cells were plated at a density of 2000 cells per well in 96-well plates in triplicates. Absorbance was measured at 565 nm using the CLARIOstar multiplate reader (BMG Labtech, Champigny-sur-Marne, France) at indicated time points.

### Apoptosis Assays

After exposure to drugs at indicated concentrations and times, cells were labeled with Annexin V-PE (BD Biosciences, Le Pont de Claix, France) and 7-AminoActinomycin D (7-AAD) (BD Bioscience) and analyzed using Flow Cytometry as described.^4^

### siRNA and CRISPR/Cas9-Mediated EZH2 Targeting

Small interfering RNAs (siRNAs) targeting EZH2 were purchased from Eurofins (Ebersberg, Germany). Transfection protocol is detailed in Supplementary Methods.

For CRISPR/Cas9 mediated EZH2 KO, details are described in the Supplementary Methods.

### Label-Free Quantitative Proteomics

Three independent biological replicates of NEM157i, NEM163i, and SU-DIPG-IVi cell lines were performed to compare the following conditions: biological effects of GSK126 versus control and siRNA-EZH2 versus siRNA control. The steps of sample preparation and protein digestion by trypsin were performed as previously described^4^ and in the Supplementary data section. Proteomic data are available at http://www.proteomexchange.org, ID PXD017525.

### Migration Assays

About 2 x 10^4^ cells/well were placed in a 96-well and incubated for 24h. An IncuCyte WoundMaker (96-pin woundmaking tool) was used to make scratches (Supplementary Methods).

### DMG Spheroid Cultures

Standard culture methods for spheroid cultures were used. Details are in the Supplementary Methods section.

### DMG In Vivo Models

Animal procedures were carried out in agreement with the European (directive 2010/63/UE) and French (decree 2013-118) guidelines. Mouse experiments have been authorized by local ethic commission and validated by the French Minister of Higher Education, Research and Innovation (APAFIS #13466-2019032112211281, authorization number B33063916).

The DMG NEM157i/NEM157i-VEGF CAM model has been described before.^4^

A murine orthotopic model (SU-DIPG-IVi-Luc in NOD/LtSz-scid IL2R gamma mice) was developed based on the work of Mohammed et al.^3^ Additional details are in the Supplementary Methods.

### Statistical Analysis

Statistical analyses were standard procedures and are detailed in Supplementary Data.

All tests were two-sided.

## Results

### EZH2 Gene and Protein Expression in DMG Samples and Cell Lines

**Figure 1.**
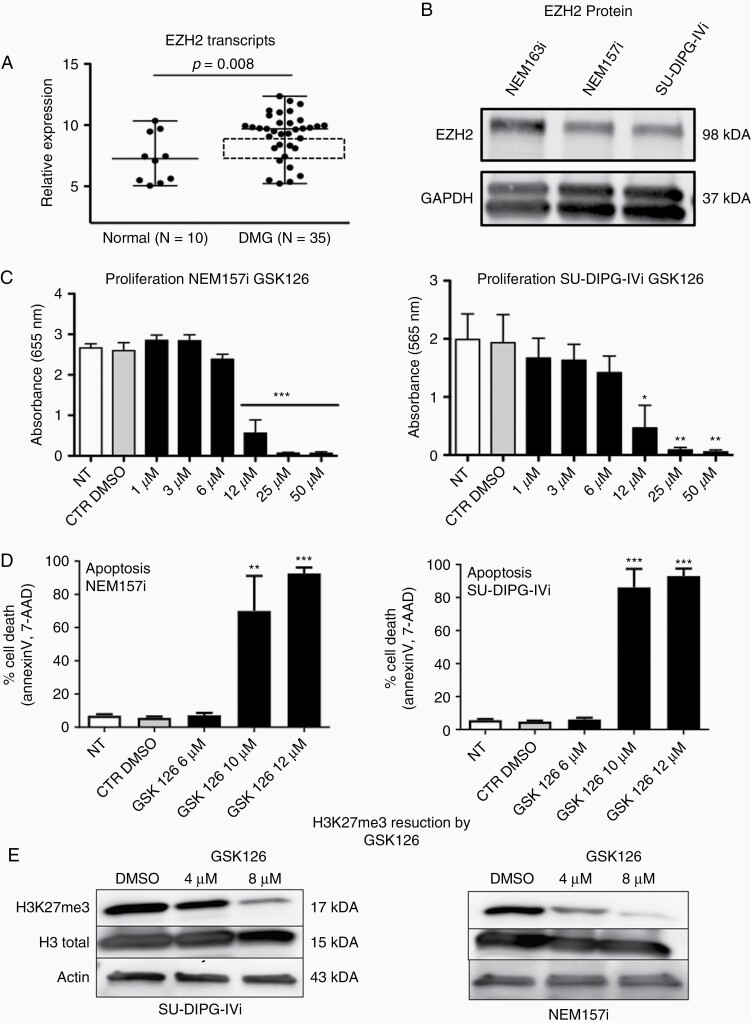
DMG cells are sensitive to GSK126 at low doses evidenced also by H3K27me3 trimethylation. (A) EZH2 transcripts are significantly overexpressed in a series of DMG biopsies (*N* = 35) compared to normal brain (*N* = 10), microarray data retrieved from GSE50021 [12]. Dashed square delimits samples regrouped around median expression. Parametric *t* test between DMG biopsies versus normal brains B) Patient-derived cell lines express EZH2 protein detected by Western Blot. (C–D) GSK126 strongly inhibits proliferation of NEM157i and SU-DIPG-IVi DMG cells after 72h and induces tumor cell death after 5 days of treatment. One way ANOVA (*n* > 3, *P* < .0001), Bonferroni’s multiple comparisons post-test. (E) H3K27me3 trimethylation is reduced a dose-dependent manner in GSK126 treated NEM157i and SU-DIPG-IVi DMG cells. One blot representative of 3 independent experiments. *P < .05; **P < .01; ***P < .001.

Transcripts of EZH2 are significantly over expressed in DMG samples compared to normal brain (Figure 1A; ^13^). Mean EZH2 over expression was not very elevated compared to controls due to high expression variability between the two groups, but a core set of samples regrouped around the median EZH2 expression and differences are still significant (Figure 1A, left plot, boxed frame). Cell lines used in this study show all expression of EZH2 protein as revealed by Western Blot (Figure 1B).

Significant growth inhibition of GSK126 was observed at doses above 6 μM and was total at higher doses above 25 μM (Figure 1C). Growth inhibition was accompanied by an increase in tumor cell apoptosis, at similar doses when cell proliferation was strongly reduced (Figure 1D). Typical H3K27me3 reduction after GSK126 treatment is revealed by Western Blot in two DMG cell lines (Figure 1E).

### Proteomic Analysis of EZH2 Impairment by GSK126 or siRNA

Volcano plot expression comparison of the two EZH2 inhibition approaches already revealed profound differences (Supplementary Figure 1). Whereas chemical inhibition upregulated 55 proteins in at least two out of three cell lines (Supplementary Figure 2A, left panel), only one protein was upregulated in the same cells after EZH2 knock down (Supplementary Figure 2A, right panel). These data point to profound biological differences caused by these two methods which cannot be explained by simple EZH2 function interference.

We submitted 55 upregulated proteins to gene ontology tools (PANTHER) to identify biological processes enriched in GSK126 treated cells. Importantly, a very significant enrichment (up to >100 times) of genes involved in the cholesterol biosynthesis pathway was evidenced (Supplementary Figure 3). In fact, 11 out of the 55 proteins upregulated by the treatment play key roles in lipid metabolic processes (Supplementary Figure 2B). These results suggest that profound metabolic changes have occurred in GSK126-treated DMG cells.

### Inhibition of EZH2 Expression by siRNA and CRISPR/Cas9 and Growth Consequences in DMG Cell Lines

To get a more complete understanding of EZH2 role in DMG cell growth we reasoned that knock down or genetic deletion of EZH2 could produce similar results. However, use of a very efficient siRNA which reduced EZH2 protein levels almost to nothing in two DMG cell lines, produced no effects on DMG cell growth 3 or 4 days after transfection. Cell lines devoid of EZH2 continued to proliferated normally, thereby confirming that EZH2 is not required for DMG cell proliferation (Supplementary Figure 5).

Growth inhibition of GSK126 was observed after 24h or 48h in EZH2 KO cells, with IC50s comparable to normal cells controls (under 10 μM, Supplementary Figure 6A-C). Growth promoting or inhibiting roles of EZH2 have already been a debate in other type of cancers and may be due to compensation effects of EZH1.^14^

### Effects of ACSS2 Inhibitor, Atorvastatin, and Terbinafine on DMG Cells Alone or in Combination with GSK126

In order to interpret these changes, we hypothesized that several key enzymes involved in the biosynthesis of cholesterol became important to GSK126-treated DMG cells. These enzymes are known for a long time to be implicated in diseases related to lipid metabolisms.^15^ For each cholesterol biosynthesis inhibitor tested, no effects have been observed on two different tumor cell lines alone (Supplementary Figure 4).

**Figure 2.**
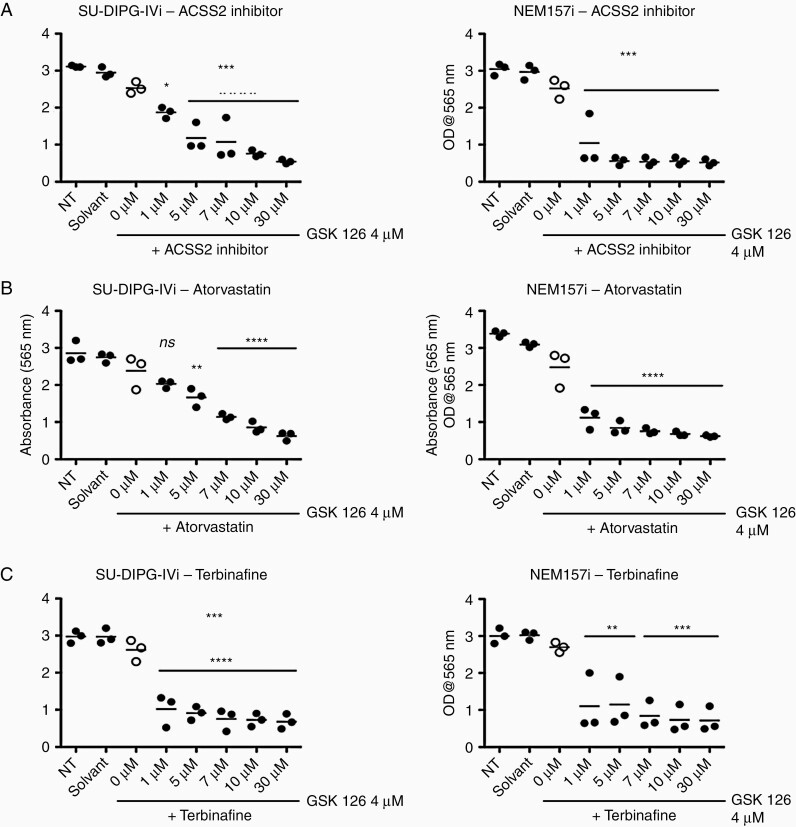
Synergistic effects of the combinatorial therapy of GSK126 and inhibitors of cholesterol biosynthesis pathway enzymes on DMG cells. (A–C) Cell growth assays of NEM157i and SU-DIPG-IVi cells (as indicated) exposed to 4 µM of GSK126, a dose which has no growth inhibitory effects on DMG cells (open circles), and to increasing doses of ACSS2 inhibitor A), Atorvastatin B) or Terbinafine C). One way ANOVA (*n* = 3, *P* < .0001), Bonferroni’s multiple comparisons post-test. Bi-therapy treatment shows significant proliferation inhibition after 72h starting at low micro molar doses of statins which show no effects alone (see Figure 4 sup). Solvant: DMSO (control, CTR), **P < .01; ***P < .001.

However, when co-treated with low doses of GSK126 (4 μM) which do not affect DMG cell growth (Figure 1C), significant growth inhibition occurs for all three inhibitors at doses from 1 to 5 μM (Figure 2A–C).

### Validation of DMG Cell Line Data on Freshly Isolated DMG Cells

**Figure 3.**
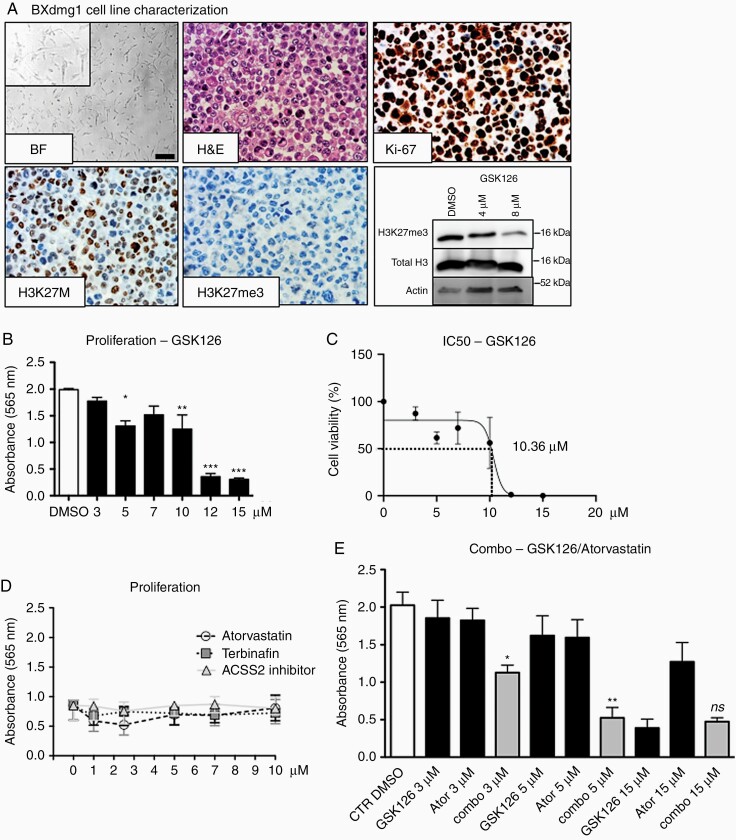
Molecular and phenotypical characteristics of the new primary DMG cell line BXdmg1. (A) BXdmg1 primary cell characterization. Upper left panel: bright field microscopy showing cell morphology of early passage BXdmg1 primary cells. Bar = 200 µm. Upper middle panel: H&E staining of Cytoblock preparation of cells revealing nuclear irregularities. Upper right panel: Proliferation status using Ki-67 staining. Lower left panel: Demonstration of presence of H3K27M mutation in BXdmg1 cells. Lower middle panel: Status of H3K27me trimethylation in BXdmg1 cells. Lower right panel: Western blot on histone protein isolation demonstrating typical reduction of H3K27me3 after exposure to GSK126. (B, C) BXdmg1 proliferation is inhibited by GSK126 after 72h with an IC50 of 10.36 µM, a comparable sensitivity to the other DMG cells. One way ANOVA (*n* = 3, *P* < .0001), Bonferroni’s multiple comparisons post-test. (D) BXdmg1 cells are not sensitive to exposure to cholesterol biosynthesis inhibitors. (E) Combo treatment of GSK126 and Atorvastatin (Ator) shows stronger growth inhibition than GSK126 or Atorvastatin alone after 72h. Combo effect is visible at low doses and is lost at higher doses of GSK126 because of its cytotoxicity alone at these concentrations. One way ANOVA (*n* = 3, *P* < .0001), Bonferroni’s multiple comparisons post-test. *ns*, nonsignificant; *P < .05; **P < .01; ***P < .001. Further information about molecular features of BXdmg1 biopsy is presented in Supplementary Figure 7.

Cell lines that are immortalized or cultured for a certain time can behave differently as the original cells. We therefore isolated cells from a DMG biopsy for functional analysis. Bright field (BF) microscopy shows a homogenous, spindle-formed cell population (Figure 3A, and insert). H&E staining of BXdmg1 cells revealed atypical eosinophilic cells with irregular anisocaryotic cell nuclei with very high mitotic activity, almost all tumor cells express the Ki-67 antigen (Figure 3A). Tumor cells were also strongly positive for the H3K27M mutation and negative for H3K27me3 trimethylation (Figure 3A). Cellular, molecular and genetic characterization of the biopsy where BXdmg1 cells originated from are summarized in Supplementary Figure 7, notably confirming the c83A>T; pK28M mutation in histone H3F3A, leading to the driver oncogenic event, which is conserved in BXdmg1 cells.

GSK126 treatment reduced H3K27me3 trimethylation in a dose-dependent manner as revealed by Western blots of histone-purified protein extracts (Figure 3A, lower right panel). BXdmg1 cells are sensible to growth inhibition by GSK126 (Figure 3B) with an IC50 around 10 μM (Figure 3C), comparable to the other DMG lines. BXdmg1 cell growth is not affected by three cholesterol biosynthesis inhibitors, up to 10 μM (Figure 3D). Combining GSK126 and Atorvastatin showed significant growth inhibition at low doses of 3 and 5 μM for each inhibitor, this effect was masked, as expected, at higher doses because of already established cytotoxicity of GSK126 alone (Figure 3E).

### Effects of Atorvastatin, GSK126, and Combination on Cell Migration

**Figure 4.**
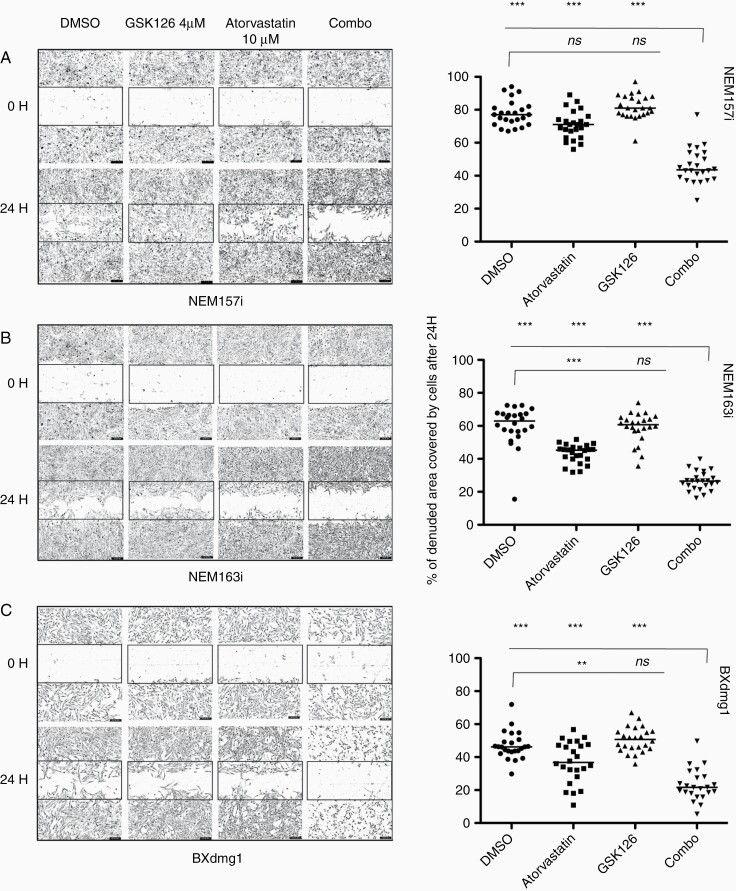
Effects of GSK126 and Atorvastatin on DMG cell migration. (A–C) Cell migration impairment in the combo treatment revealed by a wound scratch assay in the NEM157i (A) and NEM163i (B) cells and in the BXdmg1 primary cells after 24 h (C). One way ANOVA (*n* = 3, *P* < .0001), Bonferroni’s multiple comparisons post-test. *ns*, nonsignificant; *P < .05; **P < .01; ***P < .001.

We also investigated the influence of the drugs on DMG cell migration using an automated cell scratcher with Incucyte. Cell movement was measured from 24h to 48h after confluence and initiation of wound. Initial denuded area is very clean and marked by a frame of the same size for illustration in all conditions (Figure 4A–C). Percent of denuded area covered by migrating DMG cells NEM157i, NEM163i, and primary BXdmg1 cells after 24h was reported by the Incucyte software and is displayed per condition in the right graphs (Figure 4A–C). For all cells tested, a significant inhibition of migration activity (approximately 2-times) was observed for the combo treatment (*P* < .001) compared to single treatments or solvent control. In NEM163i and BXdmg1 cells, migration was also slowed by Atorvastatin compared to DMSO (*P* < .05 and *P* < .001), but cells exposed to combo treatment still had fewer area covered by migrating cells compared to Atorvastatin alone (*P* < .001). This anti-migratory effect of Atorvastatin is in line with previous results obtained in adult glioma cells.^16^

### Inhibition of Tumor Cell Spheroid Formation by Atorvastatin/GSK126 Combination

**Figure 5.**
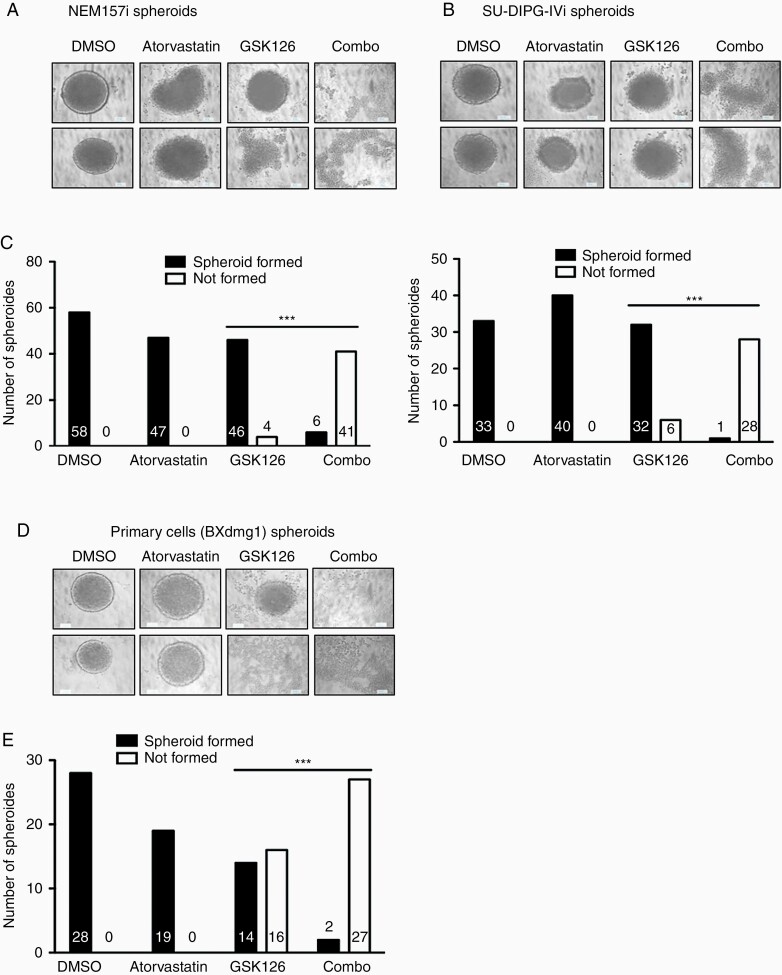
Synergistic effects of GSK126 and inhibitors of cholesterol biosynthesis pathway enzymes on DMG spheroid formation. (A, B, D) Phase contrast micrographs of representative spheroids derived from indicated DMG glioma cells after exposure to indicated inhibitors for 24 h (C, E) Statistical analysis of treatment effects on the formation of spheroids derived from the DMG cell line shown above the corresponding graph. Phenotypic criterions were spheroid formed or not formed (dispersed cells). Fisher’ Exact test was used to compare relevant treatments and the combination (combo). GSK126 dose was 20 µM for NEM157i and 15 µM for the others. ***P < .001. Time-lapse videos of inhibitor effects are demonstrated in Supplementary Figure 9 and associated videos.

In an attempt to further investigate cell movements and adhesion phenomena in our cells, we used a protocol that allows spheroid formation of DMG cells and analyzed cell movements and aggregation with an Incucyte imaging system. Tumor spheroids are considered as a more realistic culture system than classical 2D models, adding 3D complexity closer to *in vivo* growth conditions.^17^ Using three different DMG cell lines, including our primary BXdmg1 cells, we could reliably generate tumor neurospheres with all lines (Figure 5A, B, D). Round-shaped spheres formed rapidly within 24 h, albeit with slightly different sizes and sometimes differences in border shape (clear delimitated vs. irregular).

However, cells exposed to combo treatment almost never formed spheroids compared to single GSK126 treatment (*P* < .001, for all cells, Figure 5C, E, Supplementary Figure 9). In some GSK126-treated cultures, spheroid formation was affected, especially in the primary cells (Figure 5D, E).

### GSK126 Effects on Orthotopic SU-DIPG-IVi-Luc Implanted Tumor Cells in NOD/LtSz-scid IL2R Gamma (NSG) Mice

**Figure 6.**
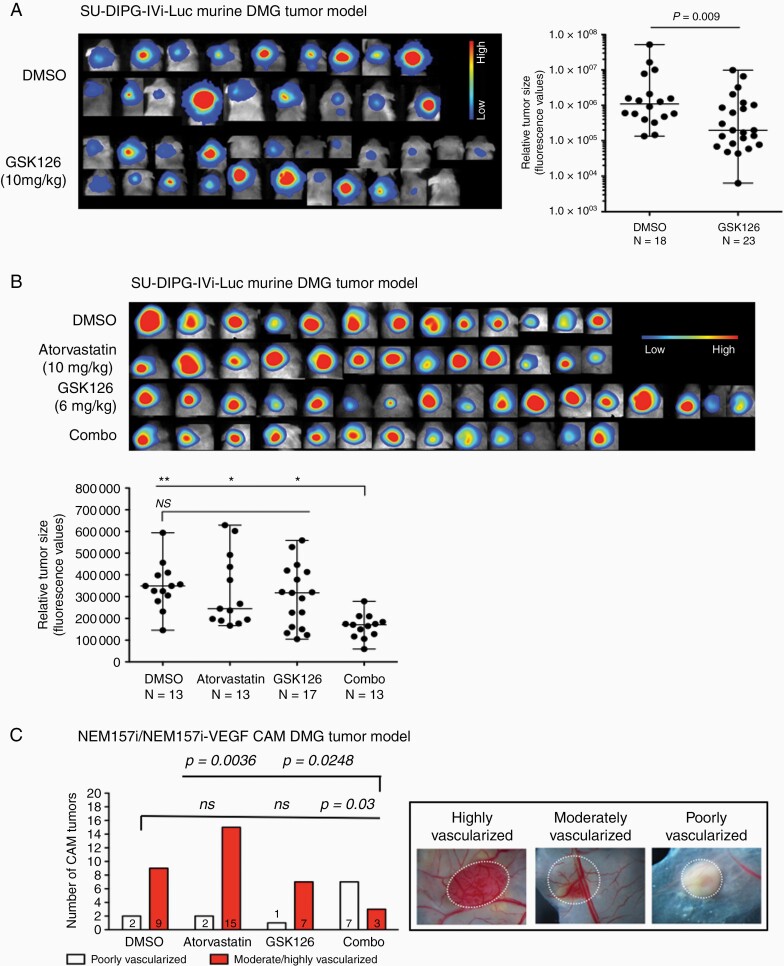
Synergistic effect GSK126 and inhibitors of cholesterol biosynthesis pathway enzymes on DMG tumor development in mice. (A) Orthotopic tumor growth inhibition of SU-DIPG-IVi-Luc cells (expressing the Luciferase) implanted in the brainstem of NOD/LtSz-scid IL2R gamma (NSG) mice after GSK126 intraperitoneal treatments. Tumor growth inhibition is demonstrated for all mice by bioluminescence live imaging and statistical analysis after the indicated treatments (solvent control: DMSO; GSK126). Color bar indicates level of expression, blue values equal low and red values high expression. Parametric *t* Student test (DMSO: *n* = 18; GSK126: *n* = 23) (B) The same approach is performed at a noncytotoxic dose of GSK126 in combination with a noncytotoxic dose of Atorvastatin. The combination of both drugs shows significant growth inhibitory effects, whereas single treatments are not effective. Bars indicate median plus range. One way ANOVA (DMSO: *n* = 13; Atorvastatin: *n* = 13; GSK126: *n* = 17; Combo: *n* = 13; *P* < .0001), Bonferroni’s multiple comparisons post-test. (C) Similar tumor growth inhibitory results were obtained using a chick CAM DMG model where a less angiogenic phenotype of implanted tumors was observed in the combo treatment, whereas single treatments had no anti-angiogenic effect Two-sided Fisher’s exact test. *ns*, nonsignificant; *P < .05; **P < .01.

We developed an ortothopic brainstem glioma model in newborn immunocompromised mice based on the work of Grasso et al..^18^ SU-DIPG-IVi-Luc cells where generated using lentiviral transfection procedures, as described (^4^ and Supplementary Methods). In a first experiment, we tested efficacy of GSK126 treatment in this model and found significant growth reduction at an i.p. dose of 10mg/kg evidenced by bioluminescence (*P* = .009) in treated mice (*n* = 23) compared to solvent controls (*n* = 18, Figure 6A).

For the Atorvastatin/GSK126 combo treatment experiment, we reduced GSK126 dose to 6mg/kg to avoid growth inhibition. After four treatments, a significantly greater tumor growth inhibition of the combo occurred (*n* = 13) compared to Atorvastatin (*n* = 13), GSK126 (*n* = 17) (*P* < .05) as well as to DMSO controls (*P* < .01, *n* = 13, Figure 6B). No significant differences have been found between controls or single treatments. All bioluminescence images of animals used in this study are shown (Figure 6A, B).

Atorvastatin/GSK126 combo treatment also exhibits better anti-tumor effects in a chick CAM DMG model we recently developed.^4^ In this short-term model, based on a previously established adult glioma CAM model,^19^ drugs can be directly applied on the tumor and growth monitored by biomicroscopy. Phenotypic characterization of drug action can be made by classifying the degree of tumor vascularization into high or low/moderate (Figure 6C, right panel). Degree of vascularization should be interpreted as capacity of tumor cells to interact with the host tissue, an indirect indicator of tumor cell aggressiveness. Combo treated experimental tumors showed reduced vascularization compared to Atorvastatin (*P* = .036), GSK126 (*P* = .0248), and DMSO controls (*P* = .03, Figure 6C, left graph).

## Discussion

EZH2 is under investigation as a potential target in cancer.^20^ However, EZH2 implication in DMG pathogenesis is still controversial. Mohammed et al. have shown EZH2 dependency of DMG cells *in vitro* and *in vivo,*^3^ whereas Wiese et al. could not evidence growth inhibition of DMG cells using two chemical EZH2 inhibitors.^21^ In the Mohammed study, the authors used first genetic engineered mouse cell lines and then a couple of H3-mutated patient lines, but not the same as in the Wiese study, except for the H3 wild-type line SF188. Whereas the first study is more focused on molecular mechanisms, the second one correlated expression levels of EZH2 to survival of patients and found no difference between high and low EZH2 expressors. So, the different results could be explained by the different approaches and cell lines used. It is likely that not all DIPG or other glioma cells do exhibit the same sensitivity to EZH2 inhibitors.

Our results suggest that EZH2 protein is not required for DMG cell growth. Neither a very efficient siRNA against EZH2 nor CRISPR/Cas9-mediated EZH2 KO in DMG cells (NEM157i and SU-DIPG-IVi) shows different proliferation patterns compared to empty control vectors (Supplementary Figure 5). Intriguingly, both knock-down and KO cells still remained highly sensitive to the EZH2 inhibitor GSK126, with an IC50 comparable to wild-type DMG cells (Supplementary Figure 6). These results strongly suggest that GSK126 affects a yet unknown growth promoting target (or targets) in DMG cells.

In an attempt to shed light on the molecular changes occurring in DMG cells treated with GSK126, we undertook a systemic proteomic approach using three different cell lines. 55 proteins were induced by GSK126 treatment with a significant enrichment of lipid and cholesterol synthesis proteins (Supplementary 2, 3).

Enzymes implicated in cholesterol synthesis have been described as potential targets in adult glioma^22^ and especially Atorvastatin has been shown to be cytotoxic in A172 human glioma cells.^16^ A recent publication by a Chinese group did find similar results as ours after treating head and neck squamous cell carcinoma with EZH2. They also observed a strong induction of cholesterol pathway genes and combination of pathway inhibitors with EZH2 augmented cytotoxic effects in these cancer cells.^23^

Recently, lanosterol synthase has been identified as a target of the menin inhibitor MI-2. Blocking this enzyme impaired biosynthesis of cholesterol in DMG, leading to glioma cell death.^24^

Deciphering the complex interactions between cancer and lipid metabolism is clearly important to elaborate novel therapeutic strategies for cancer treatment (for review see^25^).

Atorvastatin also has anti-angiogenic and anti-invasive activity^26,27^ which is in line with our CAM experiment where Atovarstatin/GSK126-treated tumors appear less vascularized and our migration and spheroid experiments which reveal significant reduction of cell motility (Figures 4, 5 and Supplementary Figure 9). These biological effects have also been shown for GSK126 in other solid tumors.^28^

We did not observe any growth inhibition of Atorvastatin alone, up to 10 μM, whereas in combination with 4 μM GSK126 (which has no inhibitory effect at that dose) significant inhibition was achieved between 1–5 μM. Similar results have been obtained with ACCS2 inhibitor and Terbinafine (Figure 2).

Combinatorial therapies are generally more effective than single treatments in complex malignant disease such as adult or pediatric high-grade glioma.^2,4,29–31^

In adult glioma, combined treatment with a dopamine receptor antagonist, quetiapine, and radiotherapy, also induces genes in the cholesterin biosynthesis pathway thereby rendering glioma cells vulnerable to atorvastatin.^29^

The combination of a statin and GSK126 seems to be a promising approach, affecting biological processes critical for tumor progression^32^ at micromolar doses at which either drug alone does not alter cell growth.

Even though significant tumor growth inhibition by GSK126 alone, and importantly, at lower doses in combination with Atorvastatin, could be evidenced in our mouse model, a more complex situation may be present in human patients. EZH2 inhibitors are prone to be excluded from penetration to the brain through specific transport efflux mechanisms implicating glycoprotein (P-gp/ABCB1) and breast cancer resistance protein (BCRP/ABCG2),^33^ but these inhibitors can achieve therapeutic concentrations in the brainstem when CED (Convection-enhanced delivery) is applied.^34^ Atorvastatin crosses the Blood-brain barrier (BBB), even though at lower levels compared to other statins such as simvastatin.^35^ Given our results with ACCS2 inhibitor and Terbinafine (Figure 2) which have not been tested *in vivo*, it is likely that brain-penetrating cholesterol biosynthesis inhibitors can be identified which can achieve therapeutic brainstem levels together with GSK126 applied by CED.

Our results did not answer all questions regarding the unexpected efficacy of the combinatory therapy, notably on cell migration and adhesion. Both processes affect cytoskeleton modifications, for example, Atorvastatin has been shown to negatively regulate cell adhesion in patients with myocardial injury^36^ and other statins have anti-migratory and anti-adhesive effects *in vitro* in melanoma cells through suppression of Rho/ROCK signaling.^37^

Epi-drugs work best in combination with other inhibitors, including chemotherapy, underlining their potential to sensitize cells to combo therapies.^38^ Atorvastatin (Lipitor®) is well tolerated in children from 6 years on^39^ and EZH2 inhibitors are currently being evaluated in clinical trials, including pediatric malignancies. Given the consistent and encouraging results presented in this paper for experimental DMG treatment, further investigation using this combination therapy should be performed, hopefully leading to clinical trials to eventually ameliorate prognosis of this dismal disease.

## Supplementary Material

vdac018_suppl_Supplementary_Figures

vdac018_suppl_Supplementary_Methods

vdac018_suppl_Supplementary_Legends
