# Supplementary material for: An EZH2 blocker sensitizes histone mutated diffuse midline glioma to cholesterol metabolism inhibitors through an off-target effect
Source: Neurooncol Adv. 2022 Mar 1;4(1):vdac018. doi: 10.1093/noajnl/vdac018 (PMC8923007; doi:10.1093/noajnl/vdac018)

Supplementary Figure 1

Volcano plots of proteome analysis of indicated cell lines and treatments

NEM157i - *DMSO vs GSK126*

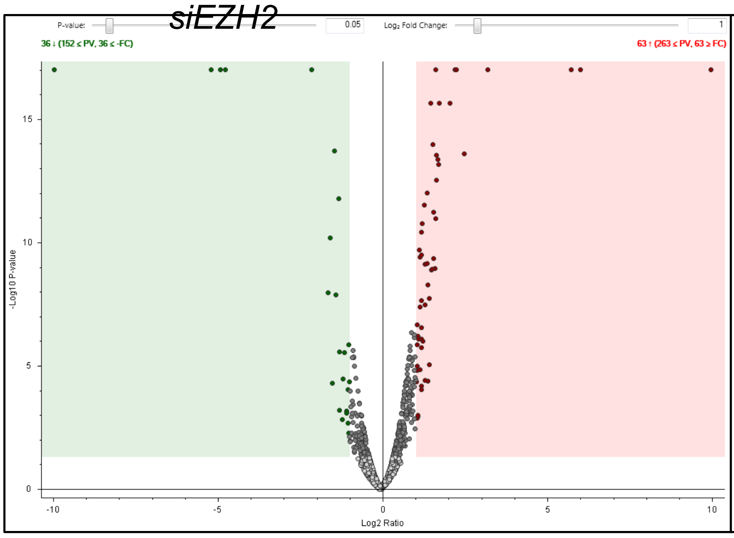

NEM157i - *siControl vs siEZH2*

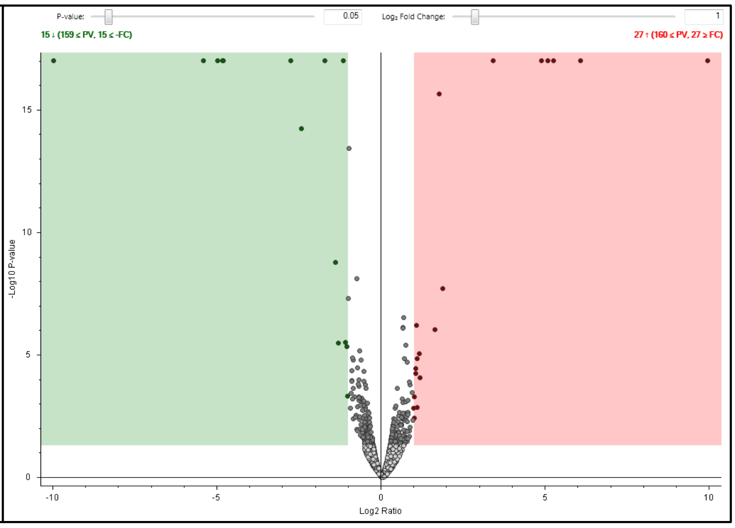

NEM163i - *DMSO vs GSK126*

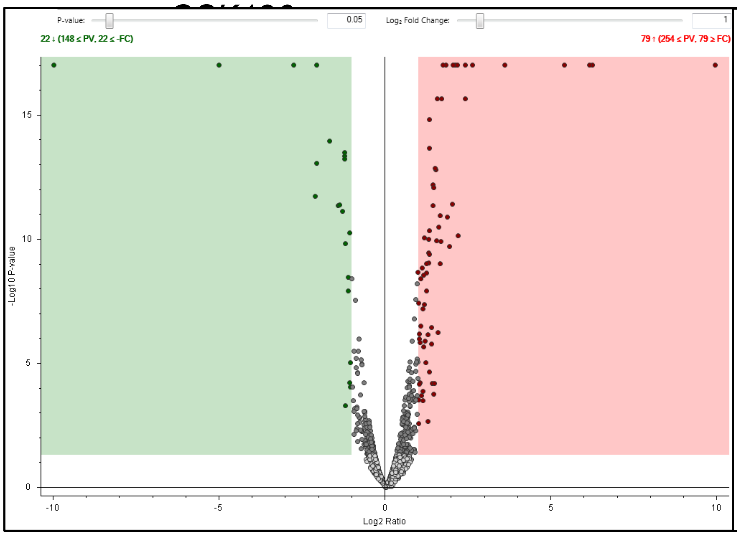

NEM163i - *siControl vs siEZH2*

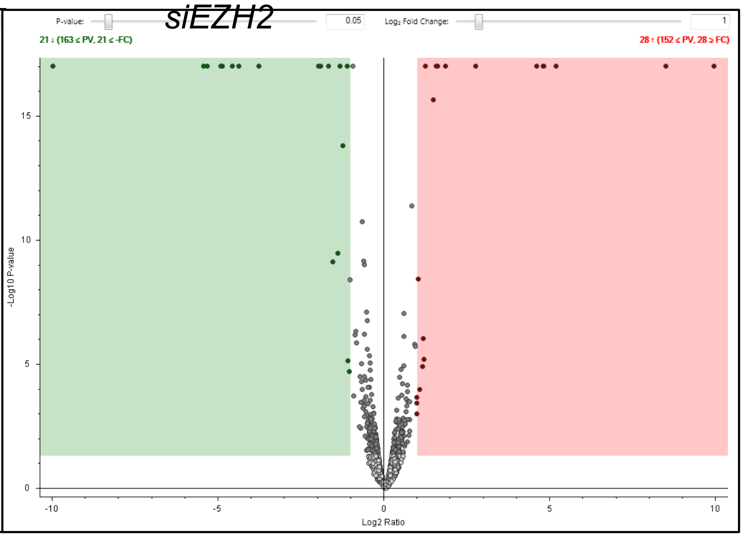

SU-DIPG-IVi - *DMSO vs GSK126*

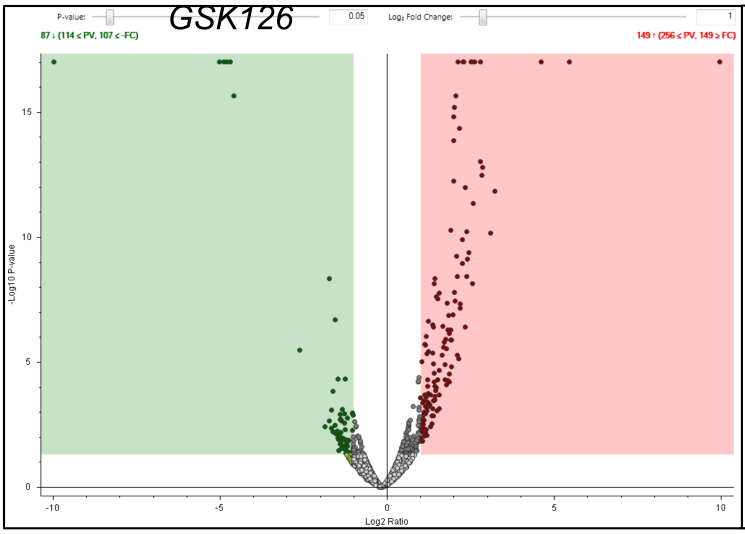

SU-DIPG-IVi - *siControl vs siEZH2*

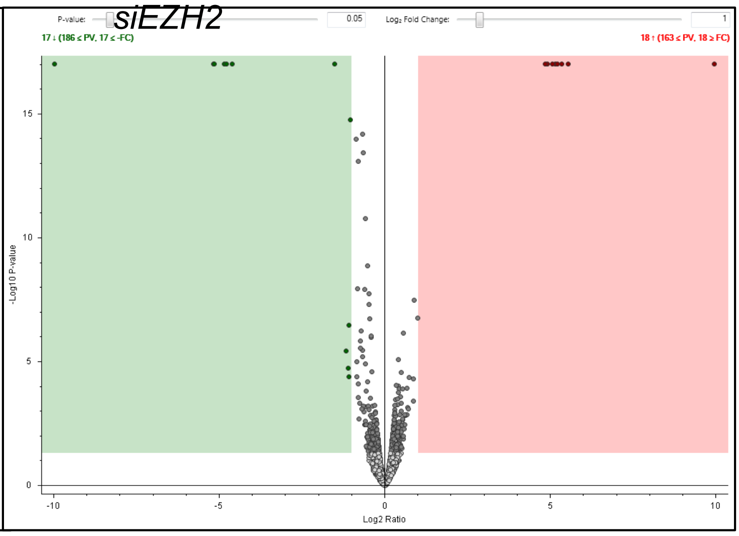

Supplementary Figure 2

A Comparisons of proteins up regulated in different cell lines after treatment

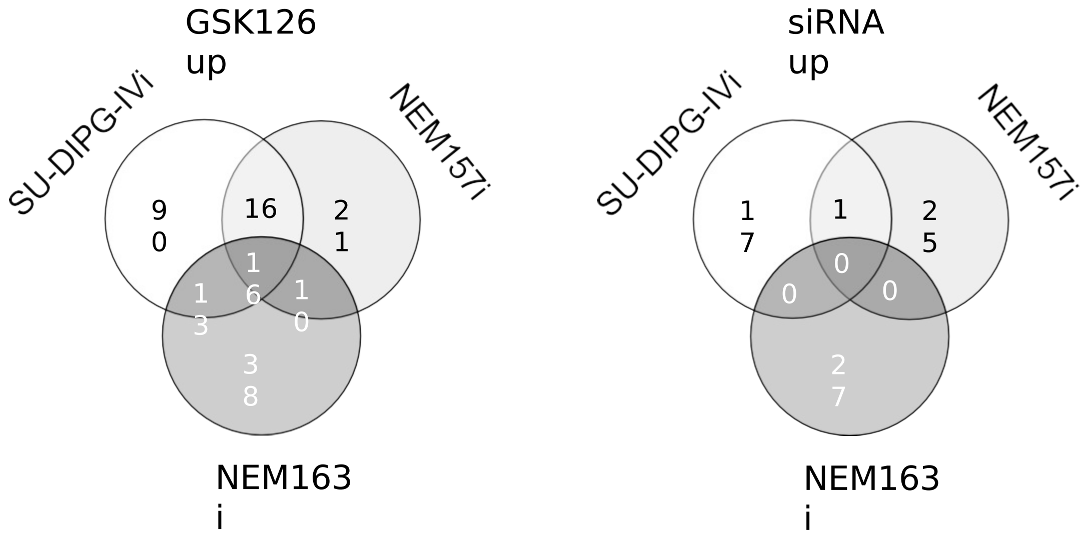

B Proteins implicated in cholesterol metabolism in at least 2 of 3 three cell lines

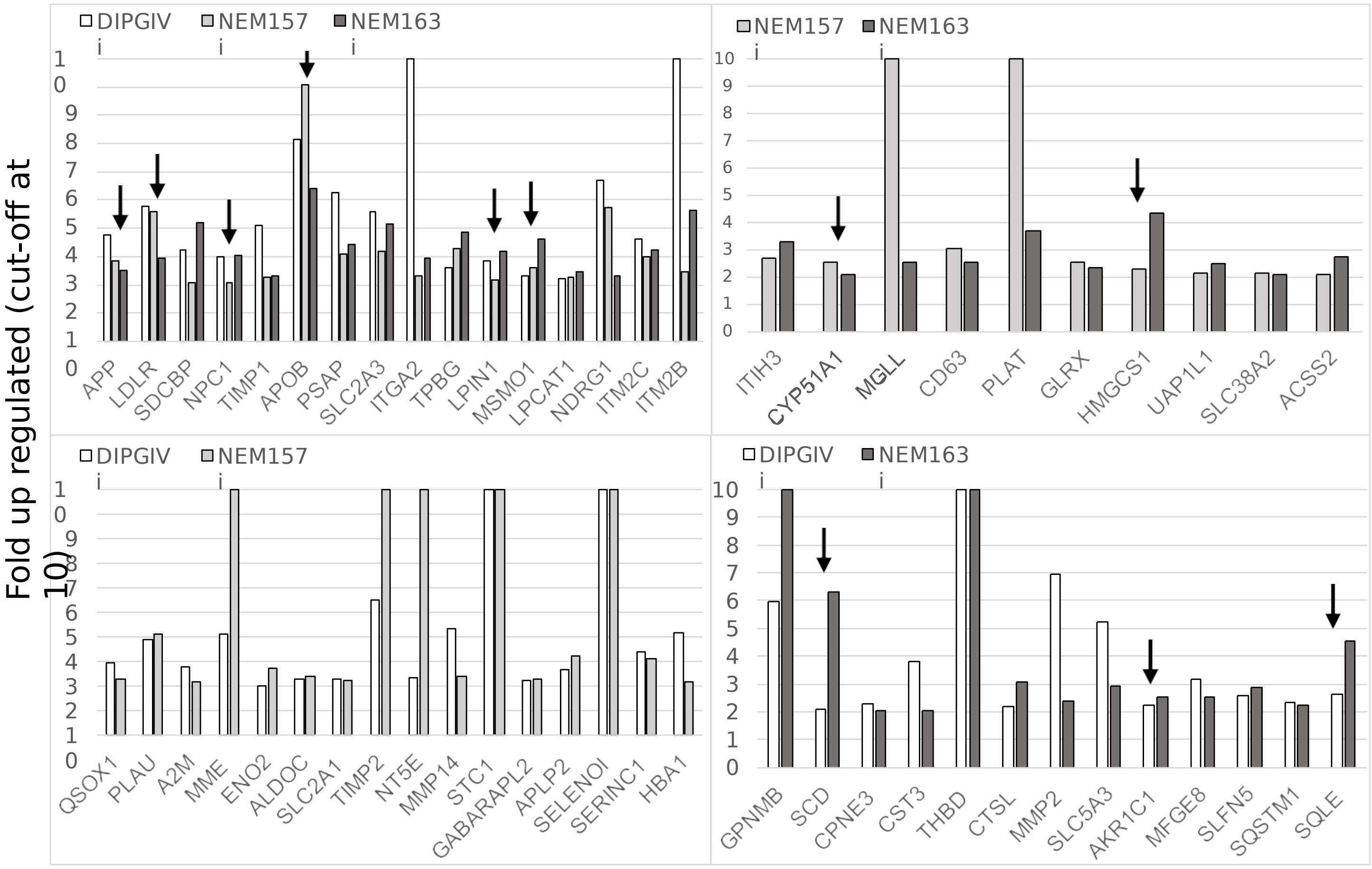

Supplementary Figure 3

Metabolic pathways enriched after EZH2 inhibition

**Analysis Type:**	**PANTHER Overrepresentation Test (Released 20190711)**					
Annotation Version and Release Date:	GO Ontology database Released 2019-12-09					
Analyzed List:	Client Text Box Input (Homo sapiens)					
Reference List:	Homo sapiens (all genes in database)					
Test Type:	FISHER					
Correction:	FDR					
**GO biological process complete**	**Homo sapiens - REFLIST**	**GSK126 induced (48)**	**No of xpected proteins**	**Pathway (fold Enrichment)**	**Raw P-value**	**FDR**
triglyceride mobilization (GO:0006642)	4	2	0.01	> 100	7.60E-05	1.44E-02
negative regulation of amyloid precursor protein biosynthetic process (GO:0042985)	7	2	0.02	> 100	1.82E-04	2.78E-02
negative regulation of metallopeptidase activity (GO:1905049)	7	2	0.02	> 100	1.82E-04	2.75E-02
negative regulation of membrane protein ectodomain proteolysis (GO:0051045)	8	2	0.02	> 100	2.27E-04	3.28E-02
smooth muscle cell migration (GO:0014909)	10	2	0.02	87	3.31E-04	4.36E-02
negative regulation of glycoprotein metabolic process (GO:1903019)	16	3	0.04	82	1.05E-05	3.16E-03
plasminogen activation (GO:0031639)	11	2	0.03	80	3.91E-04	4.94E-02
L-ascorbic acid metabolic process (GO:0019852)	11	2	0.03	80	3.91E-04	4.90E-02
glucose transmembrane transport (GO:1904659)	32	3	0.07	41	6.92E-05	1.40E-02
regulation of cholesterol biosynthetic process (GO:0045540)	43	4	0.1	41	3.95E-06	1.46E-03
regulation of sterol biosynthetic process (GO:0106118)	43	4	0.1	41	3.95E-06	1.43E-03
extracellular matrix disassembly (GO:0022617)	66	6	0.15	40	1.42E-08	2.06E-05
hexose transmembrane transport (GO:0008645)	36	3	0.08	36	9.61E-05	1.74E-02
monosaccharide transmembrane transport (GO:0015749)	38	3	0.09	35	1.12E-04	1.95E-02
carbohydrate transmembrane transport (GO:0034219)	40	3	0.09	33	1.29E-04	2.21E-02
cholesterol biosynthetic process (GO:0006695)	41	3	0.09	32	1.38E-04	2.31E-02
regulation of cholesterol metabolic process (GO:0090181)	56	4	0.13	31	1.06E-05	3.12E-03
zymogen activation (GO:0031638)	42	3	0.1	31	1.48E-04	2.45E-02
collagen catabolic process (GO:0030574)	43	3	0.1	31	1.58E-04	2.59E-02
secondary alcohol biosynthetic process (GO:1902653)	45	3	0.1	29	1.79E-04	2.83E-02
NAD metabolic process (GO:0019674)	45	3	0.1	29	1.79E-04	2.80E-02
regulation of glycoprotein metabolic process (GO:1903018)	46	3	0.11	29	1.91E-04	2.84E-02
sterol biosynthetic process (GO:0016126)	50	3	0.11	26	2.41E-04	3.42E-02
negative regulation of blood coagulation (GO:0030195)	50	3	0.11	26	2.41E-04	3.39E-02
negative regulation of hemostasis (GO:1900047)	51	3	0.12	26	2.55E-04	3.56E-02
negative regulation of coagulation (GO:0050819)	54	3	0.12	24	2.99E-04	4.07E-02
cholesterol transport (GO:0030301)	55	3	0.13	24	3.15E-04	4.18E-02
regulation of alcohol biosynthetic process (GO:1902930)	76	4	0.17	23	3.32E-05	7.66E-03
regulation of steroid biosynthetic process (GO:0050810)	88	4	0.2	20	5.74E-05	1.19E-02
cholesterol metabolic process (GO:0008203)	121	5	0.28	18	9.90E-06	3.09E-03
platelet degranulation (GO:0002576)	128	5	0.29	17	1.29E-05	3.66E-03
secondary alcohol metabolic process (GO:1902652)	134	5	0.31	16	1.59E-05	4.45E-03
negative regulation of protein catabolic process (GO:0042177)	135	5	0.31	16	1.65E-05	4.53E-03
sterol metabolic process (GO:0016125)	137	5	0.31	16	1.77E-05	4.77E-03
regulation of steroid metabolic process (GO:0019218)	118	4	0.27	15	1.72E-04	2.76E-02
female pregnancy (GO:0007565)	187	6	0.43	14	4.90E-06	1.66E-03
organic hydroxy compound transport (GO:0015850)	143	4	0.33	12	3.50E-04	4.50E-02
multi-multicellular organism process (GO:0044706)	219	6	0.5	12	1.18E-05	3.41E-03
negative regulation of cellular catabolic process (GO:0031330)	258	7	0.59	12	2.20E-06	8.75E-04
regulation of lipid biosynthetic process (GO:0046890)	191	5	0.44	11	8.24E-05	1.53E-02
alcohol metabolic process (GO:0006066)	320	8	0.73	11	7.02E-07	3.99E-04
phospholipid biosynthetic process (GO:0008654)	246	6	0.56	11	2.24E-05	5.94E-03
extracellular matrix organization (GO:0030198)	342	8	0.78	10	1.14E-06	5.68E-04
response to hypoxia (GO:0001666)	343	8	0.78	10	1.17E-06	5.63E-04
extracellular structure organization (GO:0043062)	343	8	0.78	10	1.17E-06	5.46E-04
steroid metabolic process (GO:0008202)	260	6	0.59	10	3.03E-05	7.10E-03
cellular component disassembly (GO:0022411)	394	9	0.9	10	2.75E-07	1.90E-04
neutrophil degranulation (GO:0043312)	482	11	1.1	10	1.08E-08	2.45E-05

Supplementary Figure 4

Effects of cholesterol biosynthesis inhibitors alone on DMG cell proliferation

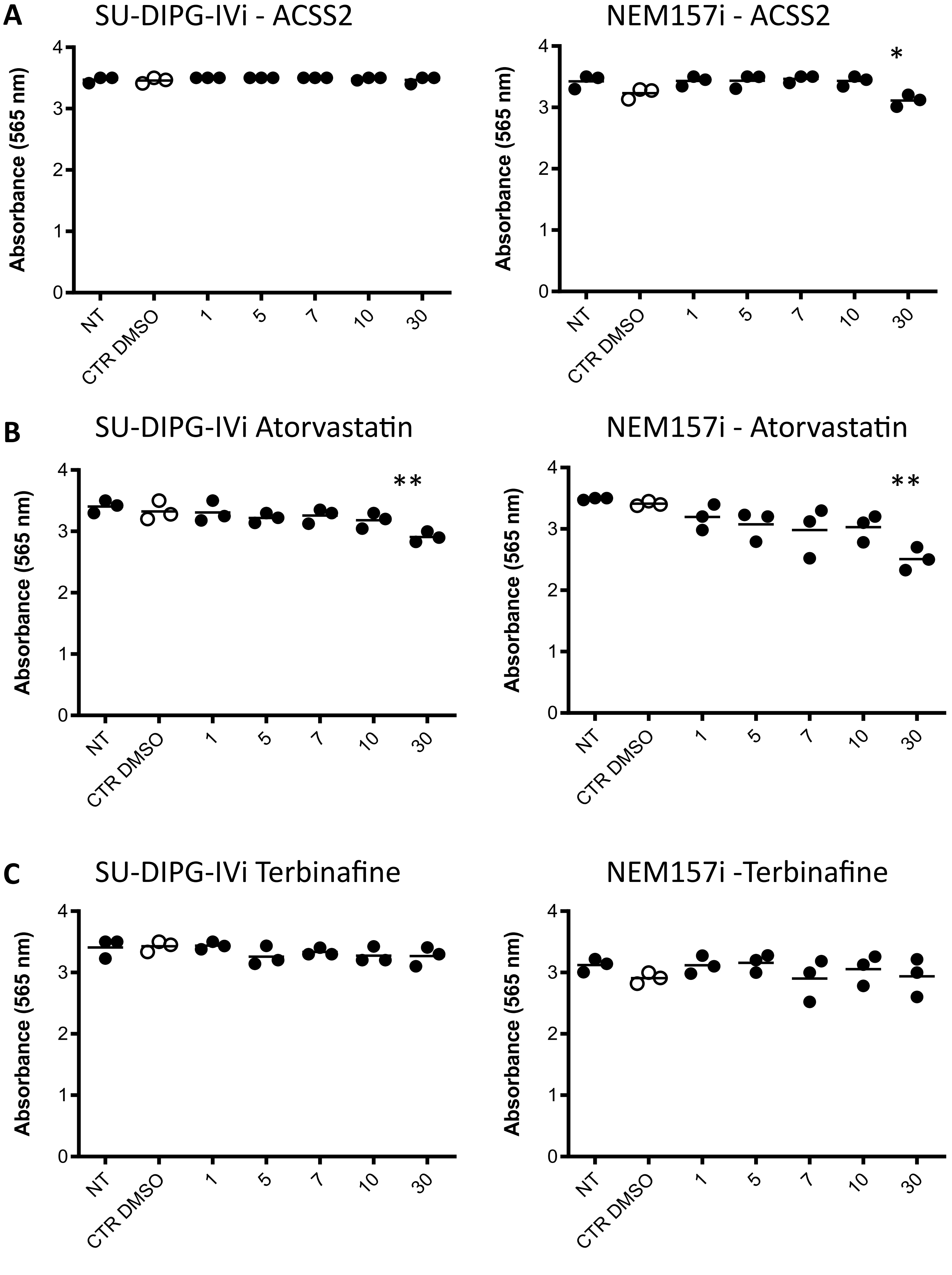

Supplementary Figure 5

Genetic inhibition of EZH2 does not affect DMG cell proliferation

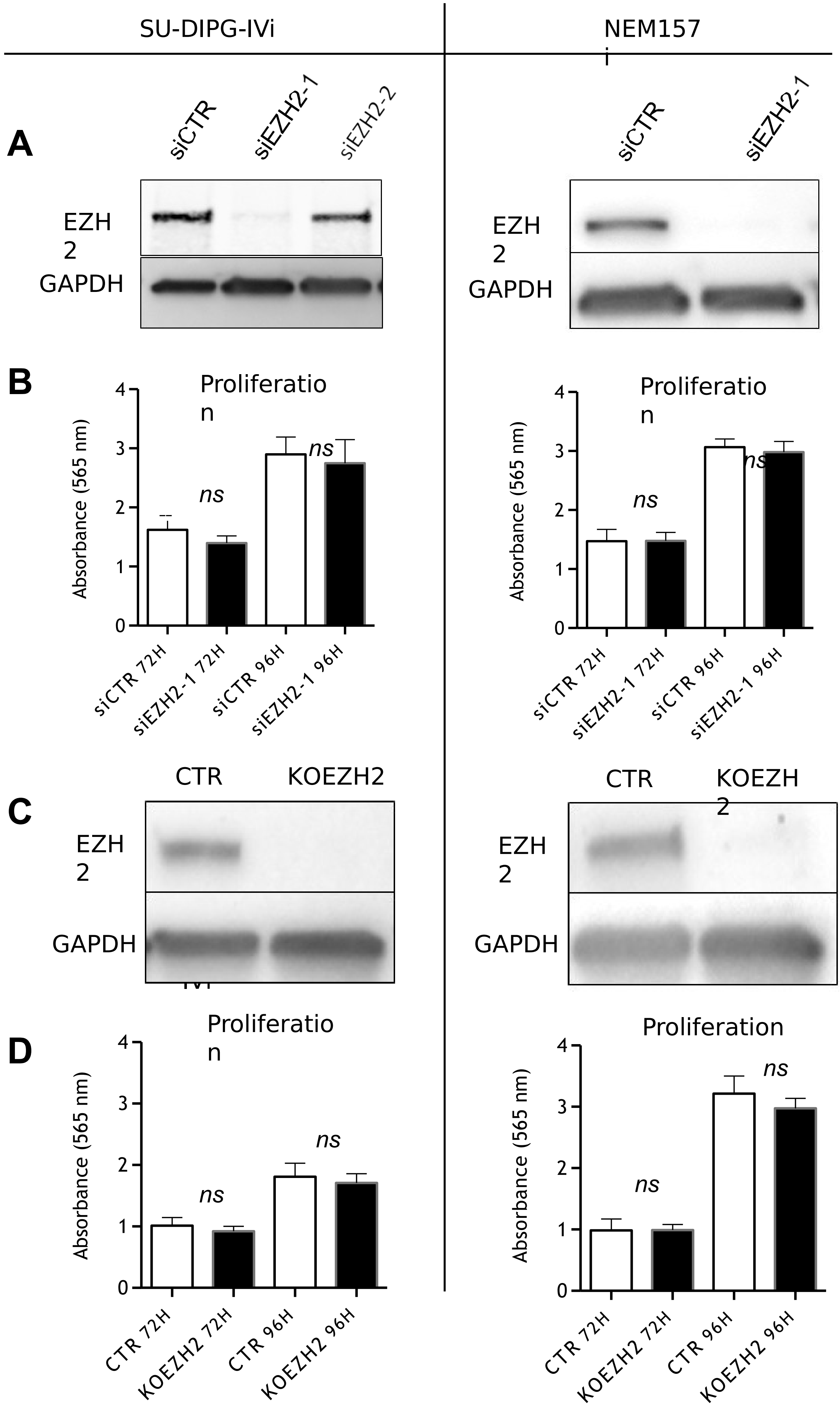

Supplementary Figure 6

GSK126 effects on DMG cells in the absence of EZH2 protein (CRISPR-Cas9 mediated KO)

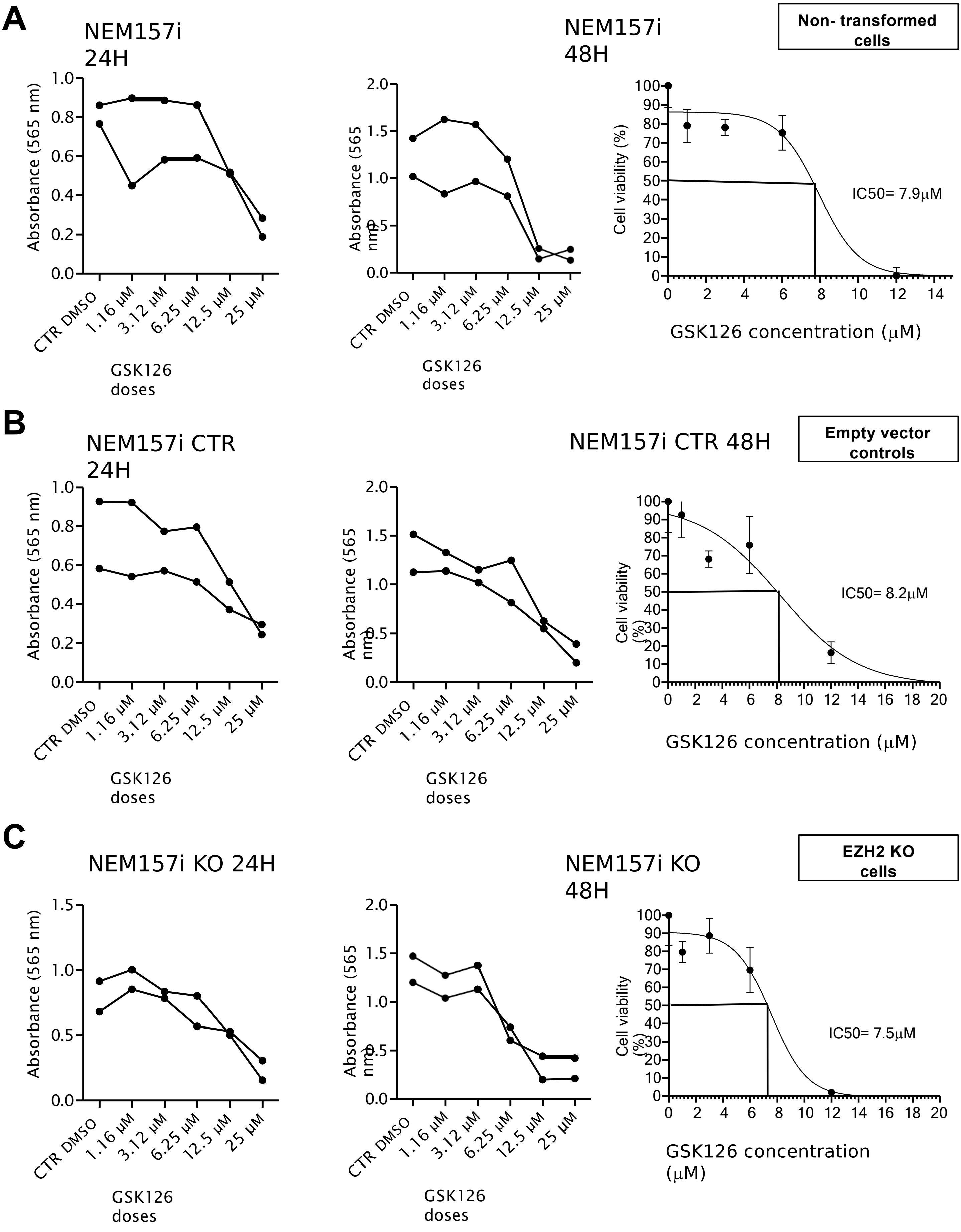

Supplementary Figure 7

A Molecular analysis of the original biopsy

High Resolution Melt (HRM)		Immunohistochemistry
No mutations	Codons OK	GFAP: 50-60%
IDH1 (exon 4, NM_005986.2)	R132	Olig2: 80%
IDH2 (exon 4, NM_002168.2)	R140 + R172	IDH1-R132H: 0%
		ATRX: 70%
Sanger sequencing		P53: >50%
Exon 2 H3F3A (NM_002107.4): c83A>t; pK28M		PS100: 0%
Exon 1 HIST1H3B (NM_003537.3): Normal	K28 normal	Synaptophysine: 0%
		PLAP: 0%
		SALL4: 0%
Fish probe 1p36/1q25 and 19q13/19p13		Proliferatin index (Mib1): 70%
1p36		EGFR not amplified
95% negative nuclei		
5% positive nuclei		
19q13		
95% negative nuclei		
5% positive nuclei		

B Analysis of 1p36 and 19q13 deletions by FISH

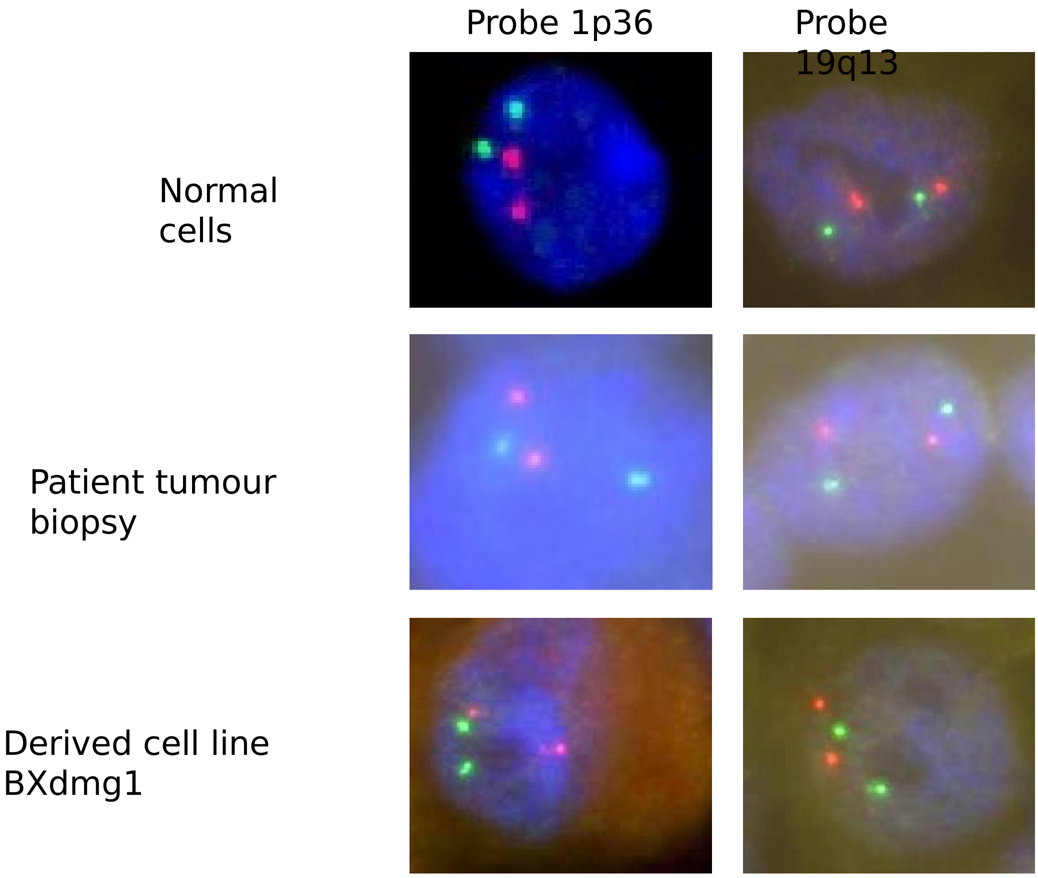

Supplementary Figure 8

H3F3A (Histone H3.3A) gene mutation analysis:
Partial H3F3A exon 2 sequence encompassing codon p.K28

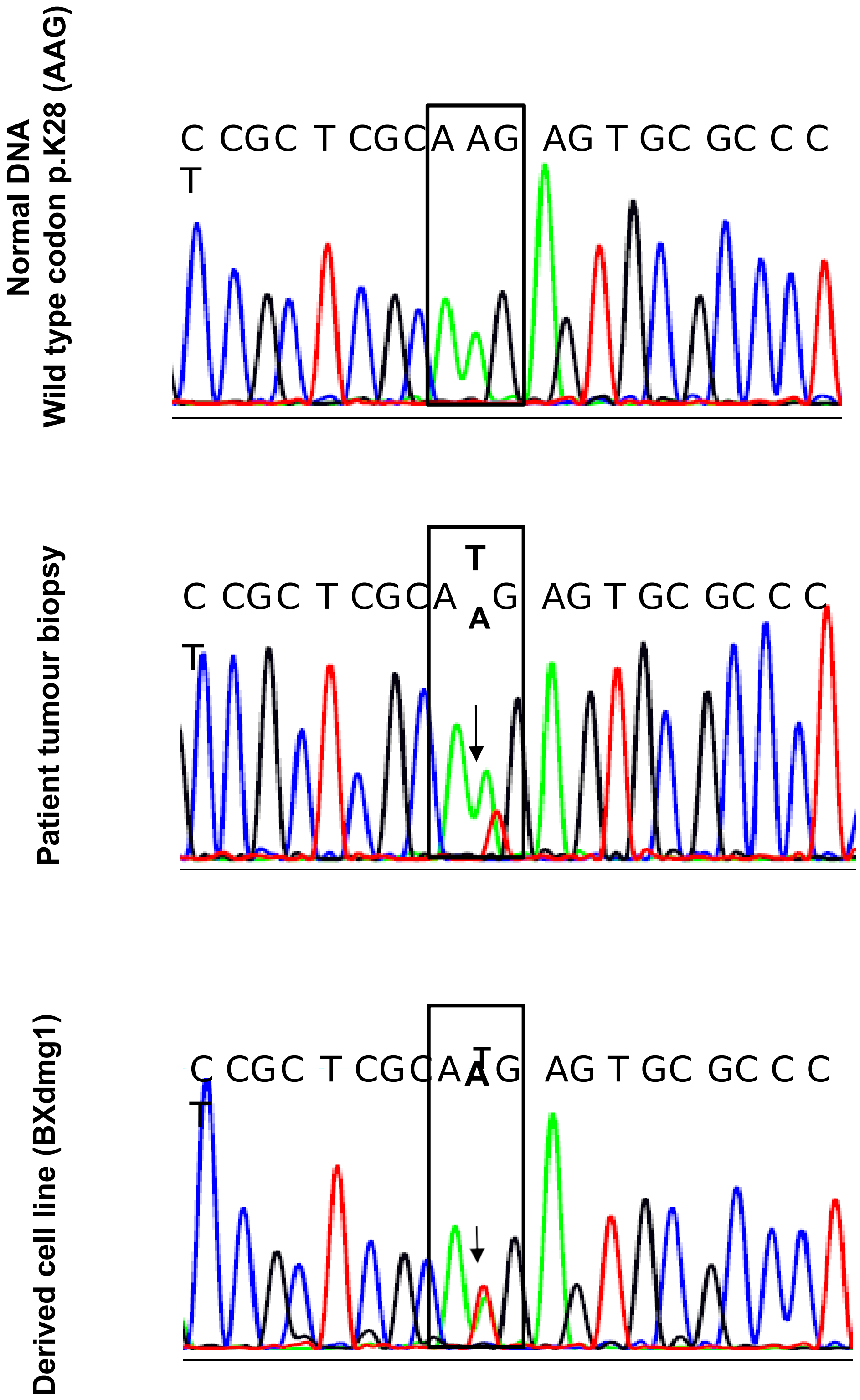

Supplementary Figure 9

Live video sequences of indicated cells during spheroid formation and treatments over 24h

(videos provided separetely)

NEM157
i

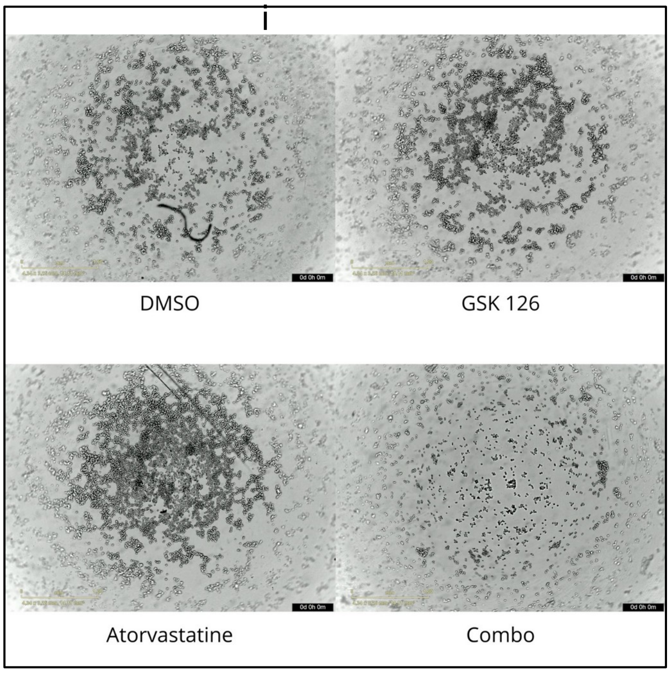

SU-DIPG-IVi

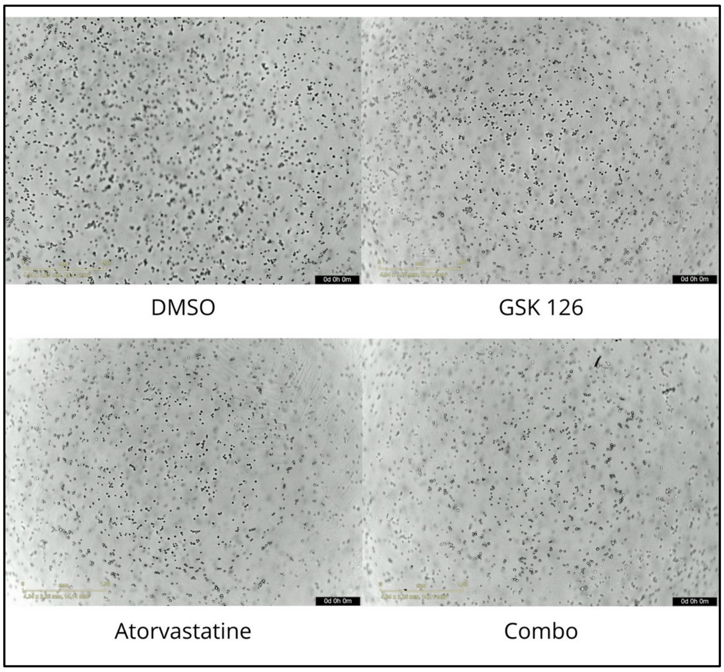

BXdmg
1

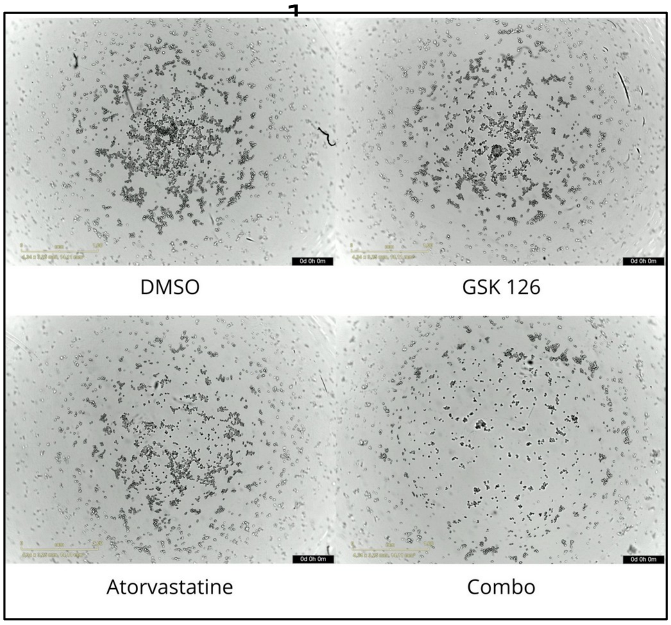

Supplement: vdac018_suppl_Supplementary_Figures [file vdac018_suppl_supplementary_figures.pdf]
