# Supplementary material for: An EZH2 blocker sensitizes histone mutated diffuse midline glioma to cholesterol metabolism inhibitors through an off-target effect
Source: Neurooncol Adv. 2022 Mar 1;4(1):vdac018. doi: 10.1093/noajnl/vdac018 (PMC8923007; doi:10.1093/noajnl/vdac018)
Supplement: vdac018_suppl_Supplementary_Methods [file vdac018_suppl_supplementary_methods.docx]

**Supplementary Methods**

**Chemical inhibitors**

GSK126 is a highly selective EZH2 methyltransferase inhibitor with an IC50 of 9.9 nM, (>1000-fold selective for EZH2 over 20 other human methyltransferases). GSK126 (SelleckChem) was dissolved with DMSO (Dimethyl sulfoxide) and stored at -20°C. Three inhibitors of the cholesterol synthesis pathway have been used: Atorvastatin, an inhibitor of HMG-CoA reductase, ACSS2 inhibitor which targets acetate-dependent acetyl-CoA synthetase 2 (ACSS2) and Terbinafine (squalene epoxidase inhibitor). All inhibitors were purchased at Selleckchemicals (Houston, USA). All inhibitors were aliquoted in DMSO and stored at -20°C.

**Primary cell isolation, culture and characterization**

All cell lines were tested regularly for the presence of mycoplasma and have been successfully STR profiled for authentication in April 2021 (LGC, Molsheim, France). Patients (or their guardians) enrolled for therapy at the University Hospital in Bordeaux gave written consent that biological probes can be used for research purposes in an anonymized manner, according to French national guidelines.

A DMG biopsy was received in the laboratory and dissociated using GentleMACs cell separator (Miltenyi Biotech, Paris, France) within 24h using MACSBrain Tumor Dissociation Kit (Miltenyi Biotech, Paris, France) and the standard GentleMACs brain tissue separation program. Cells yielded were cultivated in Amniomax C100SUP plus Amniomax C100 basal medium (Gibco, ThermoFisher, Illkirch Cedex, France). Molecular and cellular analysis of the biopsy are detailed in Supplementary Figure 6.

For characterization of biopsy-derived cells (BXdmg1) immunohistochemistry was used. Cells were harvested and included in CytoBlocks prior to sectioning and staining (Figure 5A). Automated staining was performed on an Omnis Dako® stainer after heat antigen retrieval in citrate buffer, Flex amplification treatment and incubation with following antibodies: Ki-67 (Dako, clone MIB1, 1/100), H3-K27M (Diagomics, clone RM192, 1/5000) and H3-K27me3 (ABcam, clone 8290, 1/100). Revelation was done using diaminobenzidine (Dako). The cells were regularly passaged and displayed a homogeneous morphological phenotype, they have been termed BXdmg1.

**Histone mutations of DMG cells used**

SU-DIPG-IV line is H3.1 K27M mutated (more details in Supp Table 1, Qin et al. {Qin, 2017 #4842}), NEM157 and NEM163 are H3F3A K27M (Table 1, {Truffaux, 2014 #4155}) and our BxDMG line is also H3F3A K27M mutated (Supp Figure 7, this paper)

**Western Blots**

Cell lysates were prepared by scraping cells using RIPA buffer (Sigma Aldrich) plus proteinase inhibitors (Sigma Aldrich) and centrifuged at 13000g for 15 min at 4°C. Protein concentrations were measured using PierceTM BCATM Protein Assays (ThermoFisher) and equal amounts of cell extracts were loaded for western blot analysis in 4-15 % precasted polyacrylamide gel (Bio-Rad). Proteins were blotted on a nitrocellulose membrane (Transblot® Turbo midi-size, Bio Rad), blocked with the Odyssey blocking buffer (LI-COR Biosciences, ScienceTec, Les Ulis, France) or with BSA diluted in Tris-buffered saline with 0.1% Tween 20 (TBST) and probed with primary antibodies overnight at 4°C. Primary antibodies used were: rabbit polyclonal anti-EZH2 (1:1000 dilution, #5246S, Ozyme/Cells signaling, Saint Cyr l'Ecole, France), rabbit anti-GAPDH (1:15000, BLE649203, Ozyme/Cells signaling), rabbit monoclonal H3K27me3 (1:1000, 9733S, Ozyme/Cells signaling), mouse monoclonal histone H3 (1:500, sc-517576, Santa-Cruz, Heidelberg, Germany), mouse monoclonal actin (C-2) (1:500, sc-8432, Santa-Cruz), diluted in blocking buffer or 5% BSA. After washing with TBST, the membranes were incubated with corresponding goat anti mouse IgG (H+L)-HRP conjugate (1:3000, 170-6516, Biorad, Marnes-la-Coquette, France) or anti-rabbit IgG-HRP (1:3000, A0545, Sigma Aldrich, Saint Quentin Fallavier, France). After washing with TBST (twice for 10 minutes), membranes were revealed with Fusion FX (Vilber Lourmat). Quantification was performed using ImageJ (National Institutes of Health, Bethesda, Maryland, USA).

**siRNA transfections**

siRNAs were 5′-GAGGGAAAGUGUAUGAUAA[dTdT]-3′ (siEZH2-1) and 5′-UUCAUGCAACACCCAACACU [dTdT]-3’ (siEZH2-2) and were diluted in the 1x siMAX dilution buffer (30mM HEPES, 100mM KCl, 1mM MgCl2, pH=7.3 (Eurofins, Ebersberg, Germany). DMG cell lines were transfected independently with each EZH2 siRNA or control siRNA (AllStars Negative Control siRNA, Qiagen, Courtaboeuf, France). Briefly, 10nM of siRNA was transfected with RNAiMAX transfection reagent (Invitrogen) according to the manufacturer’s instructions for reverse and forward transfections. Prior to transfection, Lipofectamine RNAiMAX (ThermoFischer) was diluted 1 / 100th in transfection medium (OptiMEM, Gibco, ThermoFisher, Illkirch Cedex, France).

**CRISPR/Cas9 system to create a EZH2 KO**

EZH2 RNA guide sequence (sgRNA) was cloned into the lentiviral vector LentiCRISPR-Cas9 521. The guide sequence was selected using the Chopchop website (https://chopchop.cbu.uib.no). Following sense (5 'CACCG ACCCCACCAAAACGTCCAGG 3') and antisense (3'CTGGGGTGGTTTTGCAGGTCC CAAA 5 ') sequences were used.

Oligo-nucleotide sequences were phosphorylated and hybridized then inserted into the linearized vector 521, by ligation using T4 ligase (NEB) at room temperature and transferred into electro-competent bacteria STBL2 (Invitrogen, Thermofisher) by electroporation then, were spread on a Petri dish containing the culture medium: LB (lysogenic broth) - agar + Ampicillin (100mg / mL). After an overnight incubation at 37°C, two clones were cultured in LB-Ampicillin and the recombinant plasmid DNA was extracted by Miniprep (Kit Plasmid DNA purification - Nucleospin plasmid [No lid], Macherey-Nagel).

The extracted DNA was digested with the restriction enzymes KpnI (NEB) and EcoRI (NEB) and deposited on 1% agarose gel in order to confirm the integrity of the restriction profile of the vector. Sequencing was then carried out to validate the insertion of the entire EZH2 guide RNA sequence into the vector 521 (Mix2Seq kit, Eurofins). The sequencing was done using the U6 primer GACTATCATATGCTTACCGT (Eurogentec). The recombinant DNA obtained by Miniprep was transformed into the XL10 gold bacteria by electroporation to amplify the recombinant DNA. After spreading the bacteria on a Petri dish, a colony was transplanted into the culture medium (LB + Ampicillin). The amplified DNA was extracted and purified by Midiprep (Kit Plasmid DNA purification - NucleoBond Xtra Midi, Macherey-Nagel).

After the production of the lentiviral vectors by transient transfection was done by the vectorology platform (Vect’UB, INSERM U1035) cell lines NEM157i and SU-DIPG-IVi were transduced with lentiviruses with different MOIs (Multiplicity of Infection). An MOI of 5 was chosen to verify the effect of the EZH2 gene deletion. The cells were then sorted by FACS in order to keep only the fluorescent cells which have integrated the recombinant vector (Cytometry platform, UB’Facsility).

**Label-free quantitative proteomics**

10µg of proteins were loaded on a 10% acrylamide SDS-PAGE gel and proteins were visualized by Colloidal Blue staining. Migration was stopped when samples had just entered the resolving gel and the unresolved region of the gel was cut into only one segment. NanoLC-MS/MS analysis were performed using an Ultimate 3000 RSLC Nano-UPHLC system (Thermo Scientific, USA) coupled to a nanospray Orbitrap Fusion™ Lumos™ Tribrid™ Mass Spectrometer (Thermo Fisher Scientific, California, USA). Each peptide extracts were loaded on a 300µm ID x 5 mm PepMap C_18_ precolumn (Thermo Scientific, USA) at a flow rate of 10 µL/min. After a 3 min desalting step, peptides were separated on a 50 cm EasySpray column (75µm ID, 2µm C18 beads, 100 Å pore size, ES803, Thermo Fisher Scientific) with a 4-40% linear gradient of solvent B (0.1% formic acid in 80% ACN) in 48 min. The separation flow rate was set at 300 nL/min.

The mass spectrometer operated in positive ion mode at a 2.0 kV needle voltage. Data was acquired using Xcalibur 4.1 software in a data-dependent mode. MS scans (m/z 375-1500) were recorded at a resolution of R = 120000 (@ m/z 200) and an AGC target of 4×10^5^ ions collected within 50 ms, followed by a top speed duty cycle of up to 3 seconds for MS/MS acquisition. Precursor ions (2 to 7 charge states) were isolated in the quadrupole with a mass window of 1.6 Th and fragmented with HCD@30% normalized collision energy. MS/MS data was acquired in the ion trap with rapid scan mode, AGC target of 3x10^3^ ions and a maximum injection time of 300 ms. Selected precursors were excluded for 60 seconds.

Protein identification and Label-Free Quantification (LFQ) were done in Proteome Discoverer 2.3. MS Amanda 2.0, Sequest HT and Mascot 2.4 algorithms were used for protein identification in batch mode by searching against a Uniprot *Homo sapiens* database (74 489 entries, release May 16, 2019). Two missed enzyme cleavages were allowed for the trypsin. Mass tolerances in MS and MS/MS were set to 10 ppm and 0.6 Da. Oxidation (M), acetylation (K) and deamidation (N, Q) were searched as dynamic modifications and carbamidomethylation (C) as static modification. Peptide validation was performed using Percolator algorithm {Käll, 2007 #4794} and only “high confidence” peptides were retained corresponding to a 1% false discovery rate at peptide level. Minora feature detector node (LFQ) was used along with the feature mapper and precursor ions quantifier. The normalization parameters were selected as follows: (1) Unique peptides (2) Precursor abundance based on intensity (3) Normalization mode: total peptide amount (4) Protein abundance calculation : summed abundances (5) Protein ratio calculation : pairwise ratio based and (6) Hypothesis test : t-test (background based). Quantitative data were considered for master proteins, quantified by a minimum of 2 unique peptides, a fold changes above 2 and a statistical p-value lower than 0.05. The mass spectrometry proteomics data have been deposited to the ProteomeXchange Consortium via the PRIDE {Perez-Riverol, 2019 #4795} partner repository with the dataset identifier PXD017525.

**Cell migration assays and spheroid cultures**

Migration was monitored by IncuCyte S3 live-cell analysis system (Essen BioScience, Ltd, Royston Hertfordshire, United Kingdom). The original unmodified Incucyte images were used for statistical analysis but for demonstration of the effects images have been transformed using Adobe Photoshop CS4 (San Jose, USA). Following functions have been used to optimize visibility of the cells: greyscale mode, bichrome mode, negative, contrast/luminosity, exposition/gamma, negative.

For spheroid formation assays, 10^4^cells were used per well in a 96-well plate. Volume of methylcellulose/medium/cells and inhibitors (10, 15 or 20 μM) mixture per well was 100 µL with a final concentration of methylcellulose at 0.5%. After rapid and gentle homogenization, the mixture was placed on a round-bottom cell culture microplate (U-shaped) treated to limit cell adhesion. After, spheroids were incubated in an incubator at 37°C and 5% CO2 for 24 hours. Photos and films of spheroids were taken by an InCellis cell imager (Bertin instrumetns, Montigny-le-Bretonneux, France). Since no dose-dependent effect was observed at the doses tested, all doses were pooled for statistical analysis.

**DMG chick and mouse models**

For the chick CAM assay DMG model, 1x10^6^ NEM157i/NEM157i-VEGF (50:50) cells were mixed with Matrigel including inhibitors at indicated concentrations and 40 µl were put directly on the CAM. Tumor growth was monitored using a stereomicroscope (DS-Fi2, Nikon/SMZ745T) every two or three days. Tumors were fixed with PFA 4% and proceeded for photo documentation.

For the mouse model, the immortalized SU-DIPG-IV line was used. SU-DIPG-IVi is transduced by a luciferase vector (Luc) with a MOI of 10. Two days after birth, 10^5^ SU-DIPG-IVi-Luc cells in 2 μl were injected directly into the brainstem through the neck to a depth of 3 mm using a 2 μl NeuroSyringe (Hamilton Neuros, Dutscher, Bruxelles, Belgium) under anesthesia with 2% isoflurane and 50% oxygen enrichment. Treatment begun after 8 days, 3-times a week with solvent control (DMSO) or GSK126 (6 or 10 mg/kg), Atorvastatin (10mg/kg) or combo (statin and GSK126 together). Tumor growth was evaluated non-invasively with anesthetized mice on a Biospace imager (Biospace Lab, Nesles la Vallée, France) 1-2 days after each treatment.

Prior to treatments, mice are micro-tattooed (Aramis kit, BiosebLab, France). Animals are weighed and then receive an i.p. injection in the lower right quadrant following IACUC recommendations. A 1 ml tuberculin syringe and a 26 gauge needle were used with an injection volume of 200μl for a 30g mouse (3 mg / ml), adjusted to actual weight each week. Treatment was done 3 times a week, starting at day 8. Tumor growth is evaluated non-invasively on a Biospace imager (Biospace Lab, Nesles la Vallée, France). The entire procedure is carried out on a heating mat at 37°C. The animals are shaved and anesthetized in sterile condition (under PSM-2) in boxes dedicated to imaging. Prior to imaging, animals receive an i.p. injection of 150mg/kg of D-Luc (Promega, E264X) diluted in PBS (50 to 100μl), depending on the weight of the animal. The box is then placed on a heating mat at 37°C in the photo imager in which 2% isoflurane with 50% oxygen is maintained. Imaging is done twice a week. Anesthesia time was around 15 minutes since only immobility is desired and awakening of the animals is almost instantaneous. At day 21, animals were killed by cervical dislocation.

**Statistical methods**

Statistical analyzes were performed with GraphPad Prism 5 software (GraphPad Software, Inc. San Diego, USA). For quantitative comparisons of more than two samples, One-way ANOVA test was used followed by Bonferroni post-test. If data distribution was not normal, Kruskal-Wallis test was used following Dunn’s post-test of selected relevant conditions. For the comparisons of two small independent samples, unpaired t-test was used. For experiments analyzed by phenotypic evaluation using a semi-quantitative approach, Fisher’s Exact test was used. All experiments (except *in vivo* experiments) were carried out, independently, at least 3 times, n=independent experiments. A *p*-value of <0.05 was considered to be statistically significant. For all data in figures, *: *p*< 0.05, **: *p*< 0.01, ***: *p* < 0.001 or exact *p*-values where indicated.
