# Supplementary material for: An EZH2 blocker sensitizes histone mutated diffuse midline glioma to cholesterol metabolism inhibitors through an off-target effect
Source: Neurooncol Adv. 2022 Mar 1;4(1):vdac018. doi: 10.1093/noajnl/vdac018 (PMC8923007; doi:10.1093/noajnl/vdac018)
Supplement: vdac018_suppl_Supplementary_Legends [file vdac018_suppl_supplementary_legends.docx]

**Supplementary figure legends**

**Supplementary Figure 1:** **Volcano plots of raw fold-change expression data of indicated comparisons**.

For each plot, on the left field, in green, protein expression is reduced, on the right in red, over expressed proteins. Chemical GSK126 inhibition and genetic EZH2 blockade are compared (left vs. right graphs) and reveal profound differences in general expression data after 48h of GSK126 treatment

**Supplementary Figure 2:** **Proteomic analysis of GSK126 and EZH2 siRNA effects on three different DMG cell lines**.

A) Diagrams indicating number of upregulated proteins by EZH2 chemical inhibition or genetic reduction of protein in three DMG cell lines, numbers in circles indicate significantly upregulated proteins and intersections indicate number of identical proteins upregulated in at least two or three out of three cell lines B). Bioinformatics analysis of shared upregulated genes in indicated cell lines which belong to cholesterol biosynthesis metabolic processes significantly over represented in GSK126 treated cells (indicated by grey circles).

**Supplementary Figure 3: Protein deregulated after GSK126 treatment**.

55 proteins common to at least two out of three cell lines were converted to gene symbols and submitted to Panther over representation test. Only significantly enriched pathways with more than 10-fold enrichment are shown. Highlighted rows indicate enriched pathways implicated in cholesterol biosynthesis.

**Supplementary Figure 4: Absence of effects of inhibitors of cholesterol biosynthesis pathway enzymes (doses shown on X-axis) on the viability of SU-DIPG-IVi and NEM157i DMG cells.**

(A-C) Three different chemical inhibitors of different enzymes implicated in the cholesterol biosynthesis pathway and induced by GSK126. A) Terbinafine: squalene epoxidase - SQLE, B) Atorvastatin: hydroxymethylglutaryl-coenzyme A (HMG-CoA) reductase - HMGSC1, C) ACSS2 inhibitor: acetate-dependent acetyl-CoA synthetase 2 - ACCS2] have no effect on DMG cell proliferation up to 30µM, after 72h suggesting insensibility of the cells to these inhibitors in the absence of GSK126. One way ANOVA (n=3, p<0.0001), Bonferroni's multiple comparisons post-test. *, p<0.05; **, p<0.01.

**Supplementary Figure 5:** **Genetic silencing of EZH2 has no effect on DMG cells**.

A) Knock down of EZH2 using siRNA (siRNA EZH2-1) completely blocks EZH2 protein expression after 72h, siRNA siEZH2-2 showed no effect. B) NEM157i and SU-DIPG-IVi cell growth over 72h to 96h did not differ between control siRNA and EZH2-1 siRNA. C) Lentiviral CRISPR-Cas9 mediated knockout of EZH2 validated by Western Blot. D) Indicated DMG cell lines without EZH2 did not proliferate differently compared to empty vector control cells. One way ANOVA (n=3), Bonferroni's multiple comparisons post-test. *ns*, non-significant.

**Supplementary Figure 6:** **Effect of GSK126 on EZH2 depleted cells**.

A) GSK126 treatment effects in NEM157i non-transformed cells. As expected, growth inhibition occurred with an IC50 of 7.9 µM. All curves represent two independent experiments. B) Growth of lentiviral transfected NEM157i cells with empty vector also was affected by GSK treatment with an IC50 of 8.2µM. C). Unexpectedly, proliferation of EZH2 KO cells also was also blocked in a similar manner as controls (IC50 =7.5µM).

**Supplementary Figure 7: Summary of genetic and immunohistologic characteristics of the original biopsy**.

A) Pathologic analysis and results of the primary biopsy, confirming a typical DMG glioma. B) Demonstration of the lack of 1p36 and 19q13 deletions in patient tumor biopsy and derived BXdmg1 primary cells.

**Supplementary Figure 8: Confirmation of mutation of histone H3 on BXdmg1 cell line**. Detection of H3F3A gene mutation, c.83A>T, p.K28M at codon K28 (AAG>ATG; Lys28Met), in patient tumor biopsy and derived cell line BXdmg1.

**Supplementary Figure 9:** Incucyte videos over 24h illustrating combo effects of indicated cells disposed in 96- well. Combo therapy clearly disrupts cell movements and lead to dispersed tumor cells unable to make cell attachments.
